# A TCR mimic monoclonal antibody reactive with the “public” phospho-neoantigen pIRS2/HLA-A*02:01 complex

**DOI:** 10.1172/jci.insight.151624

**Published:** 2022-03-08

**Authors:** Tao Dao, Sung Soo Mun, Zaki Molvi, Tatyana Korontsvit, Martin G. Klatt, Abdul G. Khan, Elisabeth K. Nyakatura, Mary Ann Pohl, Thomas E. White, Paul J. Balderes, Ivo C. Lorenz, Richard J. O’Reilly, David A. Scheinberg

**Affiliations:** 1 Molecular Pharmacology Program, Memorial Sloan Kettering Cancer Center (MSKCC), New York, New York, USA.; 2 Immunology Program, Weill Cornell Medicine, New York, New York, USA.; 3 Tri-Institutional Therapeutics Discovery Institute, New York, New York, USA.; 4 Weill Cornell Medicine, New York, New York, USA.

**Keywords:** Immunology, Oncology, Antigen presentation, Cancer immunotherapy, T cell receptor

## Abstract

Phosphopeptides derived from dysregulated protein phosphorylation in cancer cells can be processed and presented by MHC class I and class II molecules and, therefore, represent an untapped class of tumor-specific antigens that could be used as widely expressed “public” cancer neoantigens (NeoAgs). We generated a TCR mimic (TCRm) mAb, 6B1, specific for a phosphopeptide derived from insulin receptor substrate 2 (pIRS2) presented by HLA-A*02:01. The pIRS2 epitope’s presentation by HLA-A*02:01 was confirmed by mass spectrometry. The TCRm 6B1 specifically bound to pIRS2/HLA-A2 complex on tumor cell lines that expressed pIRS2 in the context of HLA-A*02:01. Bispecific mAbs engaging CD3 of T cells were able to kill tumor cell lines in a pIRS2- and HLA-A*02:01–restricted manner. Structure modeling shows a prerequisite for an arginine or lysine at the first position to bind mAb. Therefore, 6B1 could recognize phosphopeptides derived from various phosphorylated proteins with similar amino acid compositions. This raised the possibility that a TCRm specific for the pIRS2/HLA-A2 complex could target a range of phosphopeptides presented by HLA-A*02:01 in various tumor cells. This is the first TCRm mAb to our knowledge targeting a phosphopeptide/MHC class I complex; the potential of this class of agents for clinical applications warrants further investigation.

## Introduction

The long-term clinical responses in patients with a variety of cancers after checkpoint blockade therapy has demonstrated that T cells directed against cancer neoantigens (neoAgs) arising from tumor-specific gene mutations play a crucial role in the anticancer immunity (1–3). These neoAgs are potentially immunogenic because they are not expressed in normal tissues and, therefore, not subject to central T cell tolerance. Although neoAgs have long been envisioned as ideal targets for immunotherapy, their systematic discovery and validation has only become possible with the recent expansion in sequencing whole exome and RNA from tumors. The detection of the unique coding mutations within a tumor and prediction of potentially immunogenic epitopes generated by the mutations can be predicted by in silico algorithms and confirmed with orthogonal assays in vitro (4). A small number of such neoAgs has proven to be clinically useful in unique individuals by use of adoptive T cell therapy including melanoma and epithelial cancers, and several patient-specific vaccines are being tested (5, 6). However, predicted neoepitopes that trigger bona fide antitumor immune responses in patients are still rare, and they are difficult and expensive to identify (7, 8). Importantly, the mutations are nonsynonymous and patient specific (“private”), and they can be used in only a single patient, typically. These features of neoAgs make them less suitable for wide clinical translation.

Dysregulated protein phosphorylation is a hallmark of malignant transformation that directly contributes to oncogenic signaling cascades involved in cell growth, differentiation, and survival. Phosphorylation of serine, threonine, and occasionally tyrosine residues is retained on peptides during MHC class I and class II antigen processing and presentation on the cell surface (9, 10). Therefore, phosphopeptides derived from phosphorylation of proteins in malignant cells represent an extraordinary class of tumor specific “public” neoAgs, which are widely expressed and not patient specific. TCRs to these posttranslationally modified epitopes from cancers should have escaped central tolerance during thymic selection; therefore, these antigens are promising tumor-specific candidates for future cancer immunotherapies.

A number of phosphopeptides presented by both HLA class I and II have been reported to elicit CD4 and CD8 T cell responses (11, 12). Because of their biochemical properties, most phosphopeptides bind more strongly with the HLA-A*02:01 complex than the unphosphorylated sequences (13). In addition, analysis of the phosphopeptide/HLA-A2 complexes suggested that a direct contact of the phosphate moiety with the TCR complementary-determining region 3α (CDR3α) loop is likely to occur, due to the solvent-exposed, hydrophilic nature of phosphate (13). These features make phosphopeptide/HLA-A2 complexes potentially more immunogenic and more effective for selective TCR discrimination from the unphosphorylated peptide counterparts of the phosphopeptides. Insulin receptor substrate (IRS) proteins are adaptors that link signaling from growth factor and cytokine receptors to multiple SH2-containing signaling proteins to modulate cell growth, metabolism, survival, and differentiation (14). A phosphopeptide derived from IRS2 (pIRS2 aa 1097–1105) presented by HLA-A*02:01 is a well-characterized epitope for CD8 T cells (15). pIRS2 has been detected in a wide range of leukemias and solid tumors, including hepatocellular carcinoma, melanoma, ovarian cancer, and colon cancer cell lines, but not in normal T and B cells (16). pIRS2 has been used as a cancer-specific vaccine in clinical trials in patients with high-risk melanoma (17).

Currently, phosphopeptide-targeted therapies are being exploited clinically with vaccine and adoptive T cell transfer strategies (17, 18). However, the kinetics and potency of vaccine therapies usually restrict their applications to patients with minimal disease burden. Adoptive T cell transfer and T cell receptor gene therapy are patient specific, expensive, and depend on the availability of patient-derived cells. The use of TCR mimic (TCRm) mAbs directed to intracellular tumor antigens has emerged as an alternative, “off-the-shelf” approach to traditional TCR-based therapies. As antibodies, TCRm are structurally identical to traditional mAbs and share their pharmacologic features. However, while traditional mAbs recognize conformational structures of cell surface or extracellular proteins, TCRm recognize the complex of a peptide (9–11 amino acids) derived from intracellular proteins, displayed on the cell surface within the MHC class I molecule, which is the same antigenic structure bound by TCRs. Thus, TCRm provide recognition to truly tumor-specific antigens, most of which are intracellular proteins.

Therefore, potent TCRm to phosphopeptides could offer a controllable and widely applicable therapeutic strategy directed to “public” intracellular tumor antigens. Here, we report for the first time to our knowledge the design and development of a human TCRm for pIRS2 in the context of HLA-A*02:01 molecule.

## Results

### Validation of target pIRS2 in the context of HLA-A*02:01.

Validation of the target on cancer cells was assessed by confirming T cell reactivity against pIRS2 and presentation of the epitope by mass spectrometry (MS). To test if pIRS2 induces a T cell response that has no cross-reactivity to the unphosphorylated peptide (IRS2), purified CD3 T cells from HLA-A*02:01^+^ donors were stimulated with pIRS2, and the peptide-specific response was measured by IFN-γ ELISpot assay. The pIRS2 elicited peptide-specific T cell responses that did not cross-react with the cognate native peptide (Supplemental Figure 1A; supplemental material available online with this article; https://doi.org/10.1172/jci.insight.151624DS1). These data confirm that T cells directed against pIRS2/HLA epitopes could be specific for tumor cells undergoing dysregulated phosphorylation that discriminate against nonphosphorylated IRS2 in normal cells.

We next tested the presentation of the pIRS2 on different cancer cell lines by HLA ligand isolation and subsequent MS. We choose 2 HLA-A02^+^ hematopoietic (BV173, an acute lymphocytic leukemia [ALL]; OCI-AML02, an acute myeloid leukemia [AML]) and 2 HLA-A02^+^ nonhematopoietic cell lines (MDA-MB231, a breast cancer; TPC1, a thyroid cancer) and confirmed presentation of the pIRS2-derived HLA ligand in all 4 cell lines. Additionally, consistent with published literature, a length variant of the RVA(pS)PTSGV peptide could be detected in another HLA context: RVA(pS)PTSGVK on HLA-A*03 of U937 cells (Supplemental Figure 1B).

To additionally validate these results, we also evaluated the native IRS2 protein and its phosphoprotein in a panel of tumor cell lines by using an antibody specific for the phosphorylated IRS2 protein (against pSer1100), as well as an antibody that recognizes total IRS2 protein. The A375 cell line expressed unphosphorylated IRS2, but not pIRS2. BV173, Jeko, NCEB1, and SET-2 expressed both IRS2 and pIRS2 protein (Supplemental Figure 1C).

### Selection of ScFv specific for pIRS2/A2 complex and engineering of full-length human mAb.

Single phage clones selective for the pIRS2/A2 complex were first counterscreened against WT1-RMF/A2 and native IRS2/A2 monomers (WT-IRS2) to remove clones that bind to HLA-A2 of the peptide in the complex and any clones that bind to native IRS2/A2 complex.

Remaining clones were then screened against pIRS2/A2 monomers. Twenty-five phage clones specific for pIRS2/A2 were confirmed by ELISA coated with biotinylated pIRS2/A2 complex. Clones that had unique DNA coding sequences were characterized in secondary screens by binding to live cells using a transporter associated with antigen processing–deficient (TAP-deficient), human HLA-A0201^+^ cell line (T2) alone, pulsed with pIRS2 peptide, WT-IRS2, or other control peptides. Six of 25 clones screened showed specific binding to T2 cells pulsed with pIRS2 peptide — but not to T2, IRS2, or other control peptides. The variable regions of these 6 clones were formatted as full-length human IgG1 antibodies for further characterization.

### Specificity of the 6B1 human IgG1 mAb.

**Figure 1:**
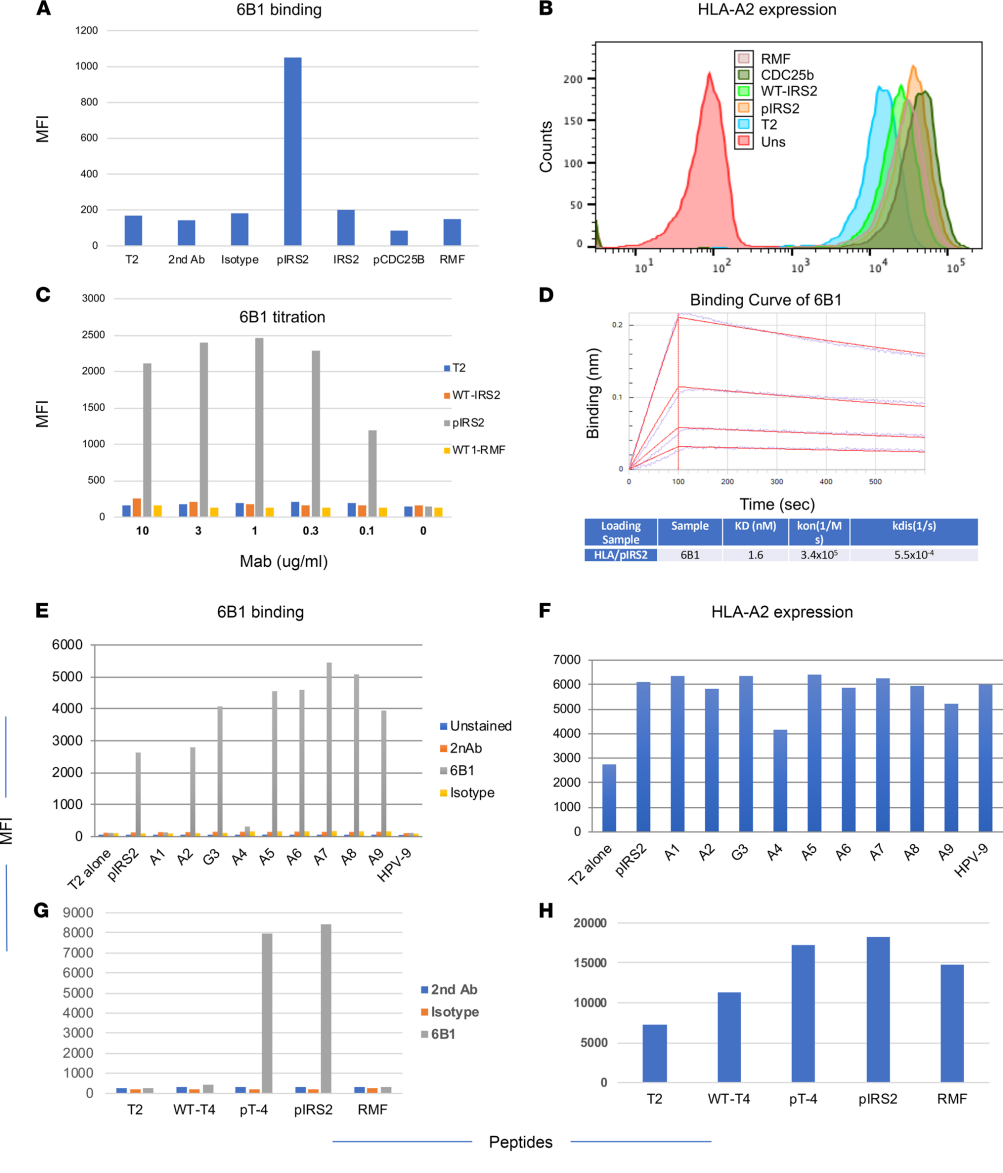
Binding of the 6B1 mAb and epitope specificity. (**A**) Binding of 6B1 to T2 cells pulsed with or without peptides. pIRS2, WT-IRS2, pCDC25b, or WT1-RMF peptide at a concentration of 20 μg/mL was pulsed onto T2 cells overnight. Cells were washed and stained with 6B1 mAb at the concentration of 3 μg/mL, followed by secondary mAb staining. The staining included secondary mAb or isotype control human IgG1. (**B**) In parallel, HLA-A2 expression was determined by staining the cells with anti–HLA-A2 mAb BB7 clone T2. Uns, unstained. (**C**) 6B1 titration was performed on T2 cells pulsed with indicated peptides and stained with indirect staining with 6B1 at concentrations ranging from 10 μg/mL to 0.1 μg/mL. (**D**) Binding kinetics of 6B1 was measured by biolayer interferometry (BLI) as indicated in Methods. The pIRS2 peptide sequence was substituted with alanine at positions 1, 2, 4, 5, 6, 7, 8, or 9 or with glycine (G3) indicated as A1–A9, and G3 and the binding of 6B1 (3 μg/mL) was determined by indirect staining and flow cytometric analysis. (**E**) T2 cells alone or pulsed with HPV peptide were the negative controls. (**F**) The same cells were simultaneously stained with anti–HLA-A2 mAb, clone BB7.2, to measure the relative binding of the peptides to HLA-A2 molecule. (**G**) Similarly, threonine substituted peptide with (pT-4) or without phosphate (WT-T4) at the position 4 was pulsed onto T2 cells and the binding of 6B1 mAb was determined by flow cytometry. (**H**) The same cells were simultaneously stained with anti–HLA-A2 mAb, clone BB7.2, to measure the relative binding of the peptides to HLA-A2 molecule.

To characterize the specificity of the full-length human IgG1 mAbs, T2 cells — pulsed with or without pIRS2, WT-IRS2, or other control peptides — initially were used to determine the binding specificity. We selected 1 hIgG1, named 6B1, out of a total of 6 mAbs based on its specificity. mAb 6B1 only bound to pIRS2-pulsed T2 cells, but not WT-IRS2 peptide and other controls, such as pCDC25b or RMF peptides (Figure 1A). Lack of binding to T2 cells pulsed with WT-IRS2, pCDC25b, or RMF was not due to poor binding of the peptides to HLA-A2 because all the peptides stabilized HLA-A2 expression (Figure 1B). Binding affinity of 6B1 was further analyzed by titrating the mAb to pIRS2 peptide. No significant changes were seen in the binding to T2 pulsed with pIRS2 peptide, down to a mAb concentration of 0.1 μg/mL, nor was significant binding observed with T2 cells alone, pulsed with WT-IRS2 or WT1-RMF peptide (Figure 1C). Affinity of 6B1 to the pIRS2/HLA-A2 complex in solution was determined to be 1.6 nM by using biolayer interferometry (BLI) kinetics assay (Figure 1D).

The phage screening strategy was designed to select clones that recognized both specific amino acids and the phosphate moiety on the serine residue, which would mimic TCR recognition. To analyze which amino acids of the pIRS2 peptide were important for recognition by the 6B1 mAb, we analyzed the binding of the mAb to T2 cells pulsed with analog pIRS2 peptides. pIRS2 peptide was substituted with alanine at positions 1, 2, 4, 5, 6, 7, 8, and 9, or with glycine at position 3 (peptides are named A1–A9, except for G3) (Table 1). The analog peptides were loaded onto T2 cells and tested for 6B1 mAb binding and for HLA-A02 expression. Alanine substitution at positions 1 and 4 strongly reduced the binding of the 6B1 mAb (Figure 1E). HLA-A2 expression was partially reduced only with substitution at position 4, but it was not significantly impaired by alanine substitution at any other positions (Figure 1F), suggesting that positions 1 and 4 were the most important residues for the mAb recognition and further demonstrating that the 6B1 mAb recognized the phosphate moiety on serine at position 4. These data support the previous studies in TCRs specific for the phosphorylated peptides bound to HLAs by which phosphorylation-generated neoepitopes discriminate their native sequences (13, 15). The mAb 6B1 showed a similar recognition pattern as the TCR for the pIRS2/HLA-A*02:01, and the native sequence was not recognized.

To further demonstrate that the phosphorylated side chain on position 4 of the pIRS2/HLA-A2 complex was important for 6B1 mAb recognition, the serine at position 4 of the pIRS2 peptide was substituted with threonine (Table 2) and tested for 6B1 recognition by pulsing onto T2 cells. mAb 6B1 did not bind to unphosphorylated threonine (WT-T4). However, 6B1 bound to the pT peptide (pT-4) at a similar level as it bound to pIRS2. These results further confirm that 6B1 recognized the phosphate moiety on position 4 of the pIRS2/HLA-A2 complex, regardless of whether it was on serine or threonine (Figure 1G). T2 stabilization assays showed that all the peptides bound to HLA-A2. It was not surprising that peptide pT-4 showed stronger binding to HLA-A2 than its native WT-T4 (Figure 1H), due to the unique feature of phosphopeptides bound to HLA-A2 molecules (13).

**Figure 2:**
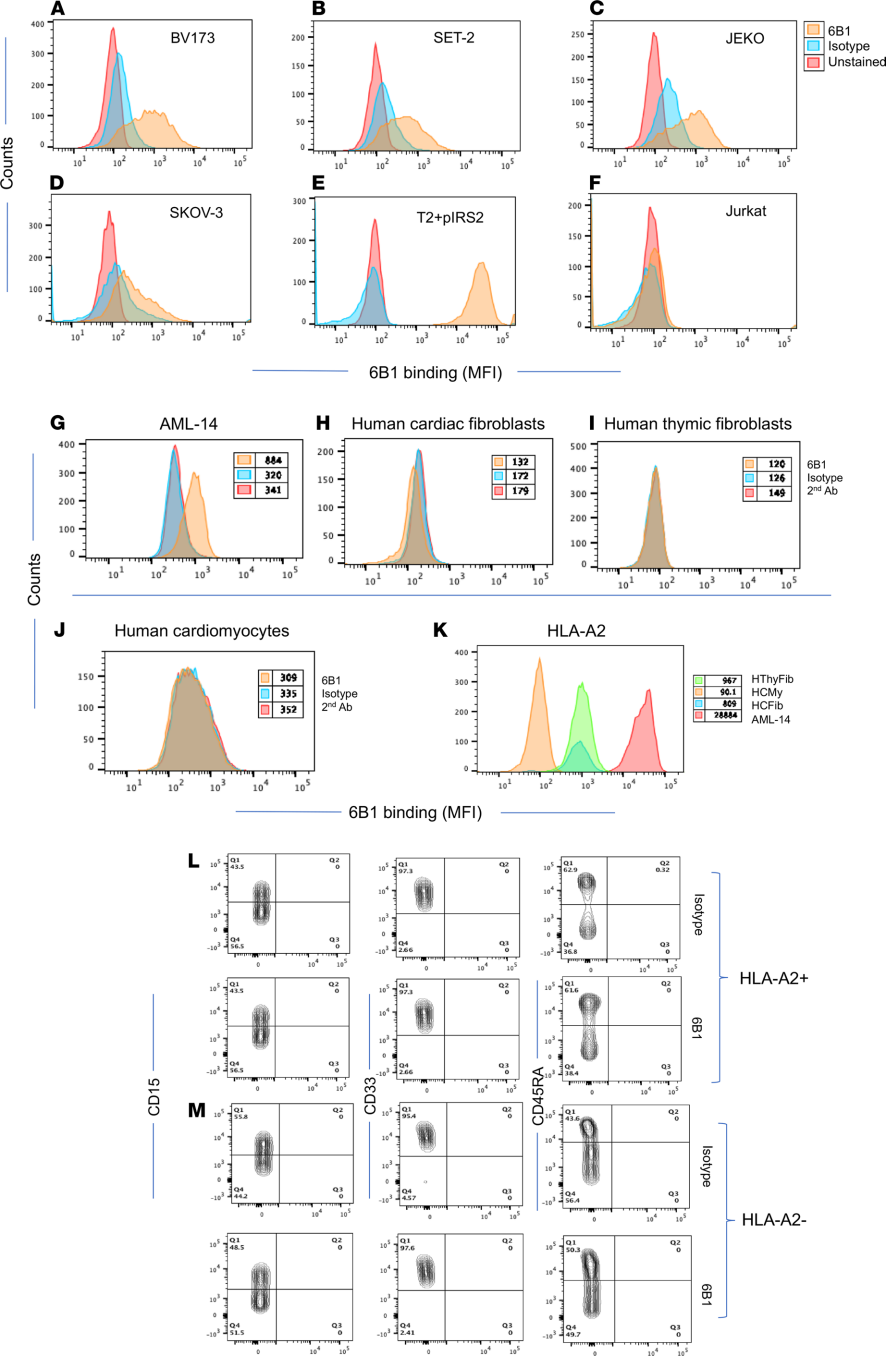
Specific recognition of tumor cells. (**A**–**F**) Recognition of the naturally presented pIRS2/A2 complex on the tumor cell surface by 6B1 in a pIRS2/HLA-A2–restricted manner was determined by flow cytometric analysis. Human leukemia cell lines BV173, SET2, ovarian cancer cell line SKOV-3 and Burkitt’s lymphoma cell line Jeko, and HLA-A2^–^ T leukemia cell line Jurkat were stained with 6B1 conjugated to APC at 10 μg/mL, followed by flow cytometric analysis. T2 cells pulse with pIRS2 was used as a positive control. Unstained cells and isotype hIgG1 were used as negative controls. Data are representative of 3 experiments. (**G**–**J**) Similarly, normal human cardiomyocytes, cardiac fibroblasts and thymic fibroblasts (**H**–**J**) and AML cell line AML-14 (**G**) were stained with 6B1 or isotype control (3 μg/mL) and followed by goat anti–human IgG Fab2 conjugated to FITC. (**K**–**M**) HLA-A2 expression (**K**) was simultaneously measured by anti HLA-A2 mAb (clone BB7.2) conjugated to APC. (**L** and **M**) Whole blood from HLA-A2^+^ (**L**) or HLA-A2^–^ (**M**) healthy donor was stained with the 6B1 or isotype control (3 µg/mL) and mAbs to CD15, CD33, and CD45RA; red blood cells were lysed; washed; and run on flow cytometry. The data represent staining from 5 separated experiments with multiple donors.

To test if 6B1 was able to recognize the naturally processed pIRS2 epitope presented by HLA-A*02:01 molecules, tumor cell lines that are HLA-A*02:01^+^ and pIRS2^+^ (15) (Supplemental Figure 1, B and C) were tested for the binding of 6B1 by flow cytometric analysis. 6B1 was able to bind tumor cell lines CML/ALL BV173, AML SET-2, mantle cell lymphoma Jeko, and ovarian cancer SKOV-3 (Figure 2, A–E). Although the pIRS2 epitope was detected by MS and Western blot in 2 other cell lines, MBA-MD-231 (breast cancer) and TPC-1 (thyroid cancer cell line), no binding of 6B1 was detected by flow cytometry (histograms not shown). It is possible that the epitope density was too low on these solid tumor cells to be detected by flow cytometric analysis. In addition, 6B1 did not bind to the HLA-A*02:01^–^ cell line Jurkat (Figure 2F). Since antigenic density of the peptide/MHC complex on the cell surface is typically 100- to 1000-fold lower than protein targets for conventional mAbs (19, 20), such low binding was expected. The results demonstrated that the mAb 6B1 was able to detect naturally presented pIRS2 epitope on tumor cells. IFN-γ treatment further enhanced the binding of the 6B1 to these tumor cell lines (Supplemental Figure 3).

To test if 6B1 recognizes the epitope on human normal cells, normal human cardiac fibroblasts; thymic fibroblasts, both HLA-A2^+^; and human cardiomyocytes (HLA-A2^–^) were tested for the binding by 6B1 mAb (Figure 2, H–K). Compared with the positive control cell AML-14, which strongly bound 6B1 (Figure 2G), all 3 of these cells were negative for 6B1 binding. The data indicate that the 6B1 does not recognize these normal cells, which most likely did not phosphorylate the peptide sequence. We further evaluated the possibility of the mAb recognizing any normal hematopoietic cells by staining whole blood with the 6B1 mAb and cell lineage markers CD15 (neutrophils), CD33 (monocytes/macrophages), and CD45 (lymphocytes) (Figure 2, L and M). 6B1 mAb did not bind to these cell populations in either HLA-A*02:01^+^ (Figure 2L) or HLA-A*02:01^–^ donors (Figure 2M).

### Antibody-dependent cellular cytotoxicity (ADCC) of mAb 6B1.

ADCC is considered to be one of the major effector mechanisms of therapeutic mAbs in humans and especially for low-density intracellular antigens such as peptide/MHC complexes (19, 20).

**Figure 3:**
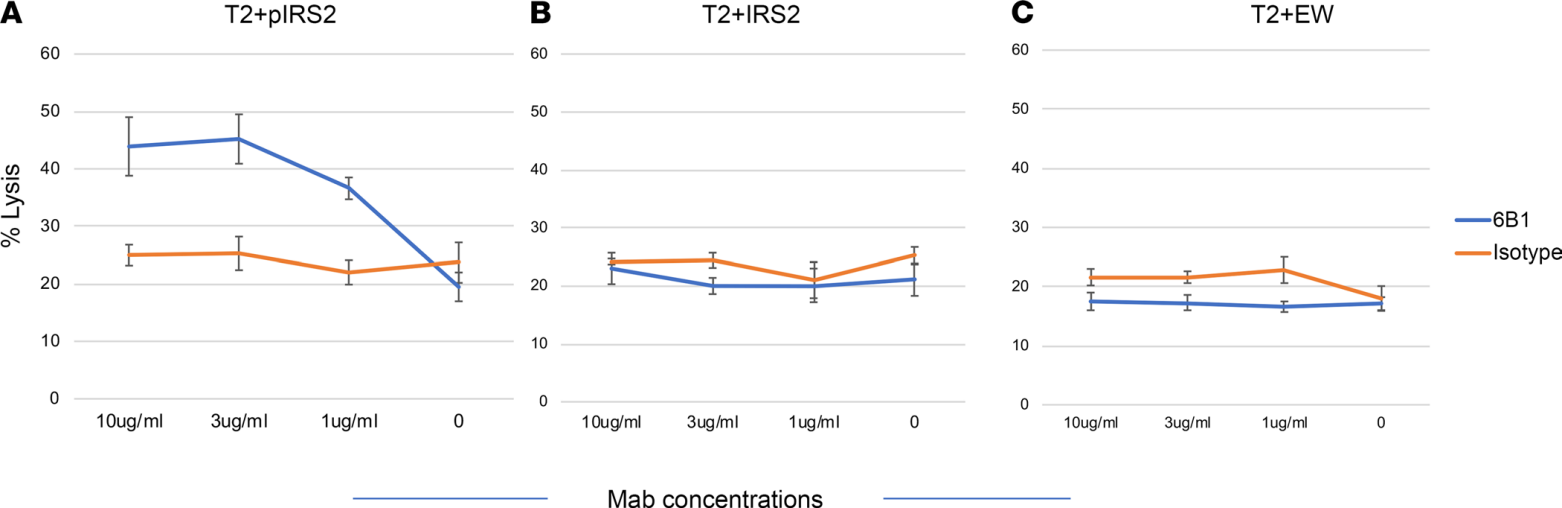
6B1 mediates ADCC with human PBMC effectors. (**A**–**C**) T2 cells alone or pulsed with pIRS2, WT-IRS2, or irrelevant EW peptides at 50 μg/mL were incubated with fresh human PBMC effectors at an E:T ratio of 30:1. Cytotoxicity was measured by 5-hour ^51^Cr-release assay. Each data point was the average of triplicate cultures ± SD; data are representative of 3 similar experiments.

Similar to its binding specificity, 6B1 was able to mediate specific ADCC against T2 cells pulsed with pIRS2 peptide, but not WT-IRS2 or irrelevant peptides (Figure 3, A–C). This demonstrated the functional cytolytic specificity of 6B1 for the pIRS2/HLA-A2 complex.

However, ADCC activity against tumor cell lines without peptide pulsing was very limited, which might be a result of the low density of the target complexes on the cell surface. To enhance its effector functions, we proceeded to engineer the 6B1 mAb into more potent formats, such as bispecific mAbs (BisAbs) engaging T cells via CD3.

### Specificity and cytotoxic function of 6B1 BisAbs.

**Figure 4:**
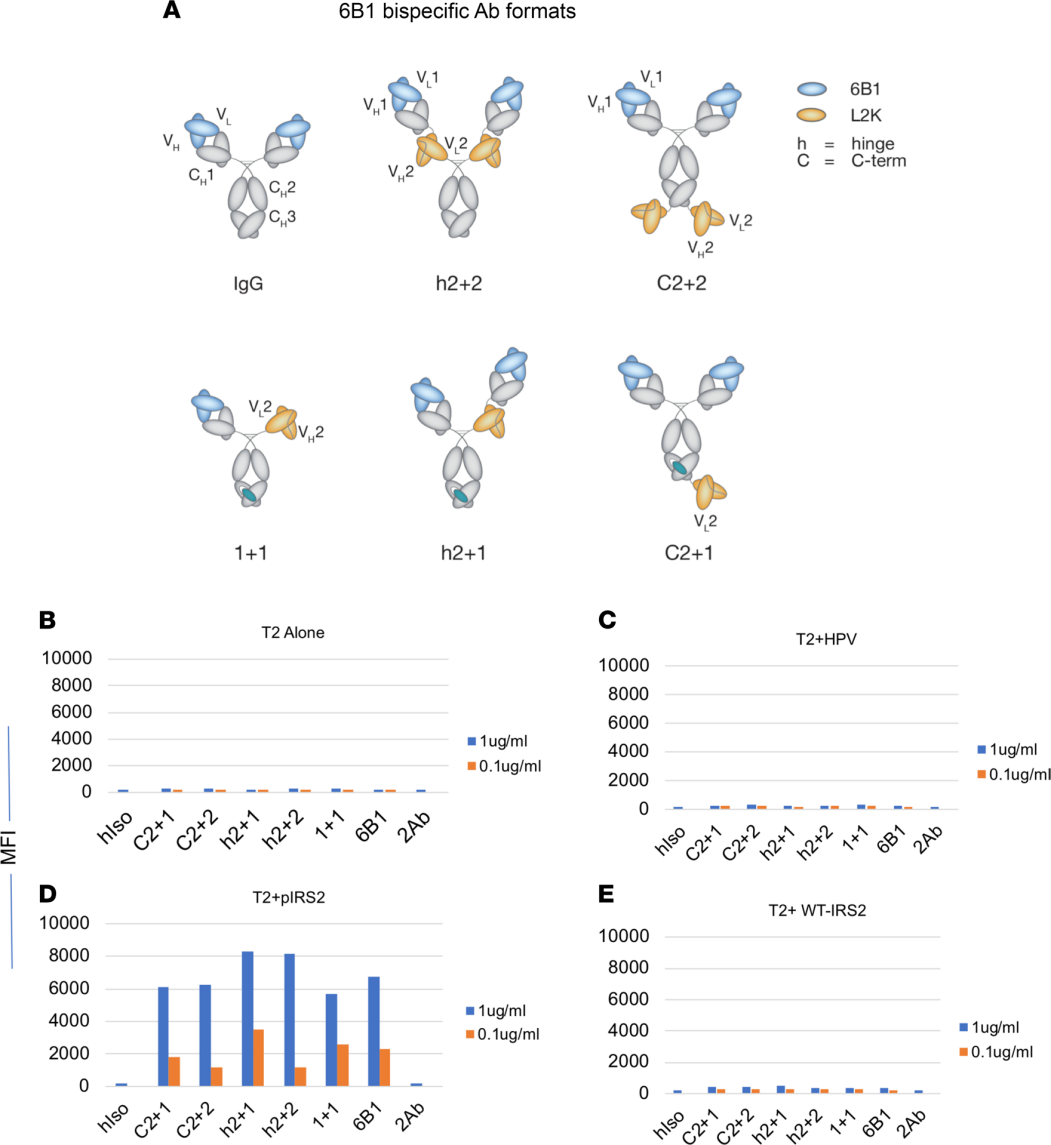
Specificity of 6B1 BisAbs. (**A**) Schematic of 6B1 BisAb panel. Five different BisAb formats engaging anti-CD3 mAb 2LK are shown as indicated. Binding of 6B1 BisAbs to T2 cells. (**B**–**E**) T2 cells (**B**) were pulsed with pIRS2 (**D**), WT-IRS2 (**E**), or HPV (**C**) peptide at a concentration of 20 μg/mL and were stained with 6B1 BisAbs at the concentration of 1 or 0.1 μg/mL, followed by secondary anti-His-tag mAb staining. The staining included secondary mAb (2Ab) or isotype control human IgG1 (hiso).

T cells have been shown to be powerful effector cells. Various formats of BisAbs engaging T cells through CD3 on T cells can bridge potent polyclonal cytotoxic T cells to the targets, by which they substantially enhance the effector functions of the IgG1 format (21, 22). To overcome the short half-life of the small BiTE molecule, which consists of 2 linked scFv fragments, we designed a series of BisAbs in human IgG formats, thus preserving the favorable pharmacokinetic profile of full-length antibodies (Figure 4A). L2K (anti-CD3 mAb) was linked to the 6B1 IgG1 format at various positions, in either monovalent (1+1, H2+1, or C2+1) or fully bivalent forms (H2+2, or C2+2).

All the engineered 6B1 BisAbs maintained their antigenic specificity for the pIRS2/HLA-A2 complex when tested for binding to T2 cells, pulsed with or without pIRS2, WT-IRS2, or HLA-A2-binding HPV E7-derived peptide (Figure 4, B–E). All 5 BisAb constructs showed binding to T2 cells pulsed with pIRS2 peptide (Figure 4D) to various degrees. No binding to T2 cells alone (Figure 4B), pulsed with WT-IRS2 (Figure 4E) or HPV peptide (Figure 4C), was observed. These results show that all BisAbs have maintained their specificity for the target antigen.

**Figure 5:**
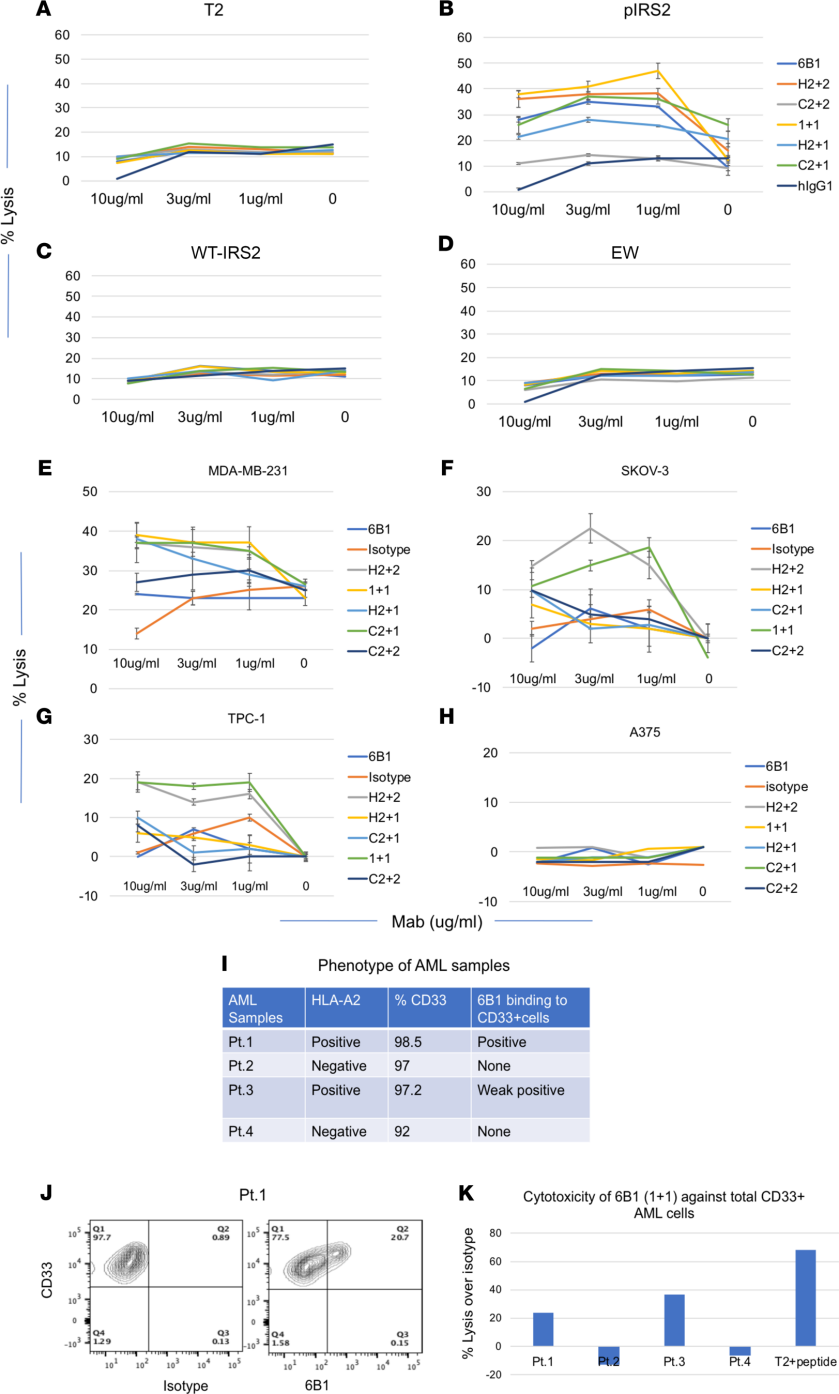
Cytotoxicity by 6B1 BisAbs. (**A**–**D**) T2 cells alone (**A**) or pulsed with pIRS2 (**B**), WT-IRS2 (**C**), or irrelevant EW (**D**) peptides at 50 μg/mL were incubated with PBMCs at an E: T of 30:1 and mAbs at indicated concentrations, and the cytotoxicity was measured by a 5-hour ^51^Cr-release assay. Each data point was the average of triplicate cultures ± SD; data are representative of 2 similar experiments. (**E**–**H**) Similarly, T cell–mediated cytotoxicity against tumor cell lines MDA-MB-231 (**E**), SKOV-3 (**F**), TPC-1 (**G**), or A375 (**H**) was measure by LDH-release assay using EBV-T cells at an E:T ratio of 20:1, with BisAbs at the indicated concentrations. Each data point was the average of triplicate cultures ± SD; data are representative of 3 similar experiments. (**I** and **J**) AML PDXs were stained with mAbs to CD33 and HLA-A2. The cells were labeled with CFSE and were incubated with activated T cells at an E:T ratio of 6:1, in the presence of 6B1 (1+1) or isotype control at 10 μg/mL. After overnight culture, cells were harvested, washed, and stained with mAb to CD33. Percentage reduction of total CFSE^+^ cells was determined as killing of the cells. (**K**) The percentage lysis of 6B1 (1+1) group was plotted over isotype control.

We next tested if the 6B1 BisAbs were able to mediate T cell cytotoxicity against the target. T2 cells pulsed with or without pIRS2, WT-IRS2, or control HLA-A*02:01-binding Ewing’s sarcoma peptide (EW), were incubated with human PBMCs used as effectors, in the presence or absence of the 6B1 BisAbs or control hIgG1 isotype (Figure 5). While there was considerable variability in potency, all 6B1 BisAbs mAb mediated specific, effective killing against T2 cells pulsed with pIRS2 peptide (Figure 5B), but not T2 cells alone (Figure 5A), WT-IRS2 (Figure 5C), or T2 cells pulsed with control peptide EW (Figure 5D). Interestingly, the C2+1 form of 6B1 BisAb was not more potent than the native hIgG1. BisAbs 6B1 1+1 and H2+2 were consistently most potent. We then tested if these BisAbs could mediate enhanced cytolytic activity against naturally presented epitopes on cancer cells. BisAbs 6B1 1+1 and H2+2 showed effective killing against all 3 cell lines MDA-MB-231, SKOV-3, and TPC-1 (which are pIRS2/HLA-A2^+^), but not against the A375 cell line, which is pIRS^–^, WT-IRS2^+^, and HLA-A2^+^ (Figure 5, E–H). The order of potency of the BisAbs was generally similar to that observed against the pulsed cell line targets. The T cell–mediated cytotoxicity against target cells BV173, SKOV-3, MDA-MB-231, Jeko, NCEB1, and SET-2 by 6B1 1+1 was further confirmed (Supplemental Figure 2). These results demonstrate that, by enhancing the effector function with T cells as effector cells, BisAbs in various formats were able to overcome the obstacle of low-target density on tumor cells, resulting in the cytotoxicity against the target. Interestingly, although all the 6B1 BisAbs should be able to recruit T cells as effector cells, depending on the design and geometry of the constructs, T cell–mediated killing activity varied significantly, which has been observed before (23–25). We next assessed the 6B1-mediated cytotoxicity against primary AML cells by using fresh frozen patient-derived samples (Figure 5I). All the samples have more than 90% CD33^+^ cells, confirming their AML cell origin. An example of 6B1 binding was shown in Figure 2J, in which 21% of CD33^+^ AML cells was bound by the 6B1 mAb. Two HLA-A2^+^ samples were variably killed by the mAb 6B1 (1+1) in the presence of activated T cells at an effector:target (E:T) ratio of 6:1, and no cytotoxicity was observed against 2 samples that were HLA-A2^–^. These data demonstrate that this BisAb is able to specifically kill the AML cells in an HLA-A2–restricted manner (Figure 5K).

### Recognition of phosphopeptides with similar amino acid compositions by 6B1.

HLA-A2 binding phosphopeptides have been shown to possess unusual characteristics. A single phosphorylated serine residue (pSer) or phosphorylated threonine reside (pThr) are located at positions 3–9, of which 68% are found at position 4. In addition, 62% of the phosphopeptides have positively charged amino acids arginine or lysine at P1; in contrast, only 9%–12% of nonphosphorylated HLA-A2 epitopes derived from either the Immune Epitope database or a set of naturally processed peptides extracted from B lymphoidblastoid cells have a positively charged amino acid at this position (13). This raised the question of whether 6B1 could recognize other HLA-A2–bound phosphopeptides with an arginine in position 1 and a phosphoserine in position 4. We selected 12 HLA-A2–bound phophopeptides to pulse onto T2 cells (Table 2). Of these peptides, 9 have arginine at position 1 and pSer at position 4; as expected, 6B1 bound to 6 of 9 peptides at different levels. No binding by 6B1 was observed in T2 cells pulsed with 3 peptides pKMD (KMDpSLDMQ), CCCK (KLIDIVpSSQKV), and β-catenin (YLDpSGIHSGA). None of these latter 3 peptides have arginine at position 1, even though pKMD and β-catenin shared the pSer at position 4. These results further demonstrate that position 1 R (arginine) and position 4 pSer are likely both required residues for recognition by 6B1. However, other amino acids in the context of the peptides also play roles in the 6B1 recognition, as the large variation in binding demonstrates.

**Figure 6:**
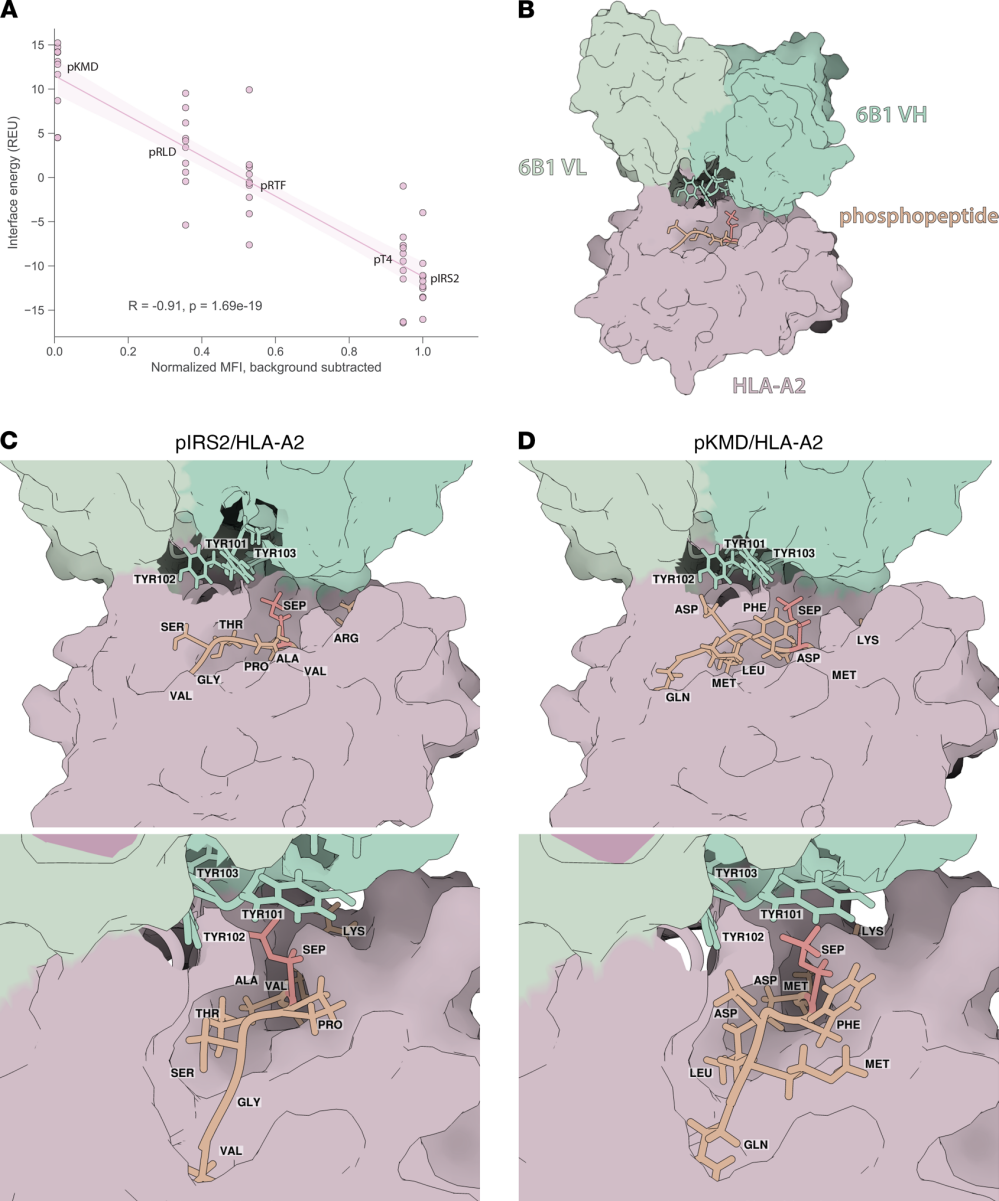
Structural modeling of 6B1 in complex with phosphopeptide/HLA-A2 complexes. (**A**) Linear regression of binding interface energy in Rosetta energy units (REU) with in vitro binding of 6B1 to the indicated phosphopeptide complexes. Regression line passes through mean of top 10 scoring models for each phosphopeptide, shaded regions represent 95% CI, and *R* value represents Pearson correlation coefficient and respective 2-tailed *P* value. (**B**) Representative model of 6B1 in complex with pIRS2/HLA-A2. 6B1 VH, VL, HLA-A2, and phosphopeptide shown in dark green, light green, mauve, and orange, respectively. Phosphoserine residue is colored red. (**C**) 6B1-pIRS2/HLA-A2 complex at different angles showing contacts of CDRH3 tyrosines (green) in contact with phosphopeptide (orange), with phosphoserine (SEP) distinguished in red. Phosphopeptide residues are labeled at their approximate locations. (**D**) 6B1-pKMD/HLA-A2 complex viewed at the same angles as in **C**.

We further investigated the variation in binding of 6B1 to different phosphopeptide/HLA-A2 complexes by computational modeling. Structural models of the 6B1 variable regions were generated using LYRA (26) and docked to HLA-A2 in complex with pIRS2, pT4, pKMD, and pRTF using the FlexPepDock web server (27) to determine the energetic favorability of 6B1 binding to each phosphopeptide/HLA-A2 complex. We selected the 10 lowest energy models for each complex and compared the energy of their respective binding interfaces, yielding a clear correlation with in vitro binding of 6B1 to each phosphopeptide/HLA-A2 complex (Figure 6, A and B). To further determine the CDR residues contributing to 6B1’s specificity, we examined the interresidue contacts between the CDRH3 of 6B1 and the phosphopeptide/HLA-A2 complex. When 6B1 is bound to its cognate pIRS2/HLA-A2, Tyr101A of its CDRH3 experiences a favorable Lennard-Jones attraction energy of –0.149 kcal/mol and Lazaridis-Karplus isotropic solvation energy (28) of 0.565 kcal/mol with pSer4 of pIRS2 (Figure 6C). When bound to pKMD/HLA-A2, Tyr101A experiences an even stronger attractive potential of –0.571 kcal/mol for pSer4 (Figure 6D); however, this is offset by a higher solvation energy of 1.259 kcal/mol. Other interresidue contacts between phosphopeptide residues and Tyr101 and Tyr102 of CDRH3 were also likely to play a role in 6B1 binding, such as the unfavorable repulsion and solvation energies of Asp7 of pKMD with Tyr102 of CDRH3 (Supplemental Table 1). Based on this computational modeling and the binding data in vitro, we concluded that 6B1 target binding is thermodynamically favorable when HLA-A2–bound phosphopeptides harbor a phosphate moiety at position 4 flanked by residues that enable favorable desolvation.

## Discussion

We designed and characterized the first TCRm directed to a cancer-associated public phosphopeptide neoAg pIRS2 in complex with HLA-A2. Phosphopeptides are emerging as a new class of cancer-specific neoAgs, which appear with dysregulated kinase activity in cancer cells. Two key features that set phosphopeptides apart from other tumor-associated antigens are: (a) TCR reactive with the phosphopeptides can discriminate them from their unphosphorylated WT counterparts, providing an increased level of cancer specificity, and (b) phosphopeptides are public neoAgs expressed in a large number of different cancers, making them distinct from patient- and tumor-specific, mutation-derived neoAgs. Strategies for T cell–based therapies to phosphopeptides have been exploited based on the role of immune responses to phosphopeptides in tumor immune surveillance (16–18, 29, 30). Recently, the first human trial using phosphopeptide pIRS2 and BCAR3 vaccines in high-risk melanoma demonstrated their immunogenicity and safety (17).

In this study, we took a potentially new approach for targeting cancer-associated phosphopeptides by developing a TCRm mAb, which provides the advantages of the well-established antibody format. We selected pIRS2 aa 1097–1105/HLA-A2 as the target, based on its wide expression pattern across hematopoietic malignancies and solid tumors, but also based on its absence in normal T and B cells (16). TCR recognition is typically focused primarily on the central amino acid residues of the HLA bound peptide. TCR recognition of phosphopeptides contributes additional specificity because of the direct contact with the phosphate moiety, typically at position 4 or 5. Our strategy resulted in the discovery of TCRm mAbs specific for the pIRS2/HLA-A2 complex that recognized the position 4 phosphate moiety, including the 6B1 mAb. In addition to the phosphoserine at position 4, we found that the 6B1 TCRm also recognized R at position 1. Phosphorylation of the IRS2 peptide may have resulted in conformational changes that allow for further distinction from the complex with the unphosphorylated IRS2 peptide (15, 27). In addition, a majority of HLA-A2 binding phosphopeptides have either arginine or lysine at position 1 that contribute to stronger binding to HLA-A2 (13). Therefore, 6B1 might accrue the conformational contributions of each of these 2 amino acid residues to the pIRS2/HLA-A2 complex (13). For TCR recognition of the phosphopeptide/HLA-A2 complex, a modeling study using superimposition of previously determined TCR-HLA-A2 complexes onto the phosphopeptide–HLA-A2 structures (PKD2 phosphopeptide: RQApSLSISV) indicated a close proximity of the CDR3α loop to the phosphate moiety, suggesting direct recognition of the phosphate moiety may occur (13). A recent study, using a soluble TCR specific for CDC25b-derived phosphopeptide GLLGpSPVRA bound to HLA-A2, demonstrated that recognition of this complex by the TCR was entirely dependent on the presence of phosphate on position 5 of pSer (28). In both studies, the TCR recognized the phosphate moiety, in addition to other respective amino acid sequences.

However, the residues at position 1 were not directly recognized by these TCRs. Since arginine at position 1 and pSer at position 4 are both crucial residues for 6B1 recognition, it was expected that 6B1 may also recognize other phosphopeptide/HLA-A2 complexes homologous at these 2 residues at position 1 and position 4, which we documented with analog peptides. Specificity analysis data also suggest that 6B1 recognition depends on individual amino acids, in addition to these 2 key residues, as has been reported for TCR recognition (27–30).

Both TCRs and TCRm mAb have significant cross-reactivities to other peptide/MHC complexes, due to their recognition of short linear peptide epitopes embedded within MHC class I–binding groove; other peptides in the exome may share amino acid homologies or physicochemical features that also allow binding (31, 32). Therefore, one of the big challenges of TCR-based immunotherapeutic approaches is to avoid off-target peptide recognition, in order to avoid harming normal tissues. Phosphopeptide-induced T cell responses did not recognize the unphosphorylated epitopes. Similarly, TCRm 6B1 did not recognize WT-IRS2, therefore discriminating against nonphosphorylated form of IRS2 and other putative nonphosphorylated peptides with similar compositions. Furthermore, 6B1 did not recognize several normal human primary cells tested, which included human cardiac and thymic fibroblasts and human cardiomyocytes. Furthermore, 6B1 did not bind to healthy donor hematopoietic cells that included neutrophils, monocytes/macrophages, and lymphocytes. This is consistent with a previous report that this phosphorylation site of IRS2 was not detected in normal hematopoietic cells (16). Before selecting a lead mAb to take forward toward clinical translation, extensive characterization as to other possible normal tissue cross-reactivities will be needed. In addition, a panel of patient-derived tumors should be assessed in vivo to determine other factors that might affect efficacy.

Recognition of phosphopeptides by these agents also might be different because in transformed cells, and dysregulated phosphorylation may generate neoAgs by affecting the shape of the presented epitopes, with phosphate participating as a direct contact element for the TCR and by altering the conformation of the peptides relative to its unphosphorylated counterpart. Most importantly, phosphorylated forms of peptides are likely to be upregulated on transformed cells, whereas unmodified counterparts may be present on normal untransformed cells. Therefore, it is possible that the recognition by 6B1 for HLA-A2–bound phosphopeptides derived from other cancer-associated proteins such as PKD2, which share similar amino acid compositions, might greatly expand the cancer target spectrum, allowing 1 mAb to target a wide array of cancers. In this regard, this pattern of recognition by 6B1 mAb could be beneficial in a therapeutic setting.

It is well known that unique peptide/MHC complexes are of far lower density than cell-surface protein antigens (19, 20, 29). In addition, many cancers can downregulate their cell-surface HLA providing resistance to TCR-based therapies. To increase the potency of mAbs targeting these antigens, bispecific antibody formats have been developed (21–25). We do not have quantitative data from MS of pIRS2 density on the surface of each tumor cell line. However, since human IgG1 formats of 6B1 did not show sufficient cytotoxicity against tumor cell lines, we engineered 6B1 into different BisAb formats, with the best cytotoxicity achieved with the with 6B1 1+1 and H2+2 formats. Although the H2+2 format can produce bivalent binding to both tumor antigen and CD3, rendering potentially higher avidity for such a low-density antigen, surprisingly, the 6B1 1+1 with monovalent binding showed comparable or superior cytotoxicity. These results are in agreement with other studies that suggest that distance and orientation between 2 targets is also important in the efficacy of the immune synapse formation for BisAbs. The proximity effects are well documented for other BisAbs (21, 23–25), and low-density targets can still be effectively utilized. The scarcity of studies using BisAb formats for TCRm mAbs limits our understanding of the full mechanisms underlying the efficacy of BisAbs for peptide MHC antigens.

In summary, this report demonstrates that it is possible to generate a TCRm mAb recognizing public phosphopeptide/HLA complexes and that the bispecific full-length mAb formats (1+1 and H2+2) can effectively enhance T cell cytotoxicity against these low-density tumor targets. More work is required to further understand the recognition patterns and potential for translation of TCRm mAb against this potentially new class of tumor antigens — in particular, therapeutic efficacy in vivo.

More potent forms of the mAb may be needed as described by others to achieve significant therapeutic effects (33). The study opens the possibility of therapeutic targeting of phosphopeptide/HLA complexes by using TCRm mAbs.

## Methods

### Cell samples, cell lines, and antibodies

Normal human cardiomyocytes, cardiac fibroblast and thymic fibroblasts were purchased from Science Cell Research Laboratories. The sources of the hematopoietic and solid tumor cell lines (Table 3) were described previously (19– 21). mAbs against human HLA-A2 (clone BB7.2) conjugated to FITC (catalog 561107) or allophycocyanin (APC; catalog 561342), and its isotype control mouse IgG2b/FITC (catalog 565379) or APC (catalog 565381), were purchased from BD Biosciences. Goat F(ab′)2 anti-hIgG conjugated with phycoerythrin (PE; catalog PA1-84341) or FITC (catalog A24465), mouse anti–human CD3 mAb (catalog 12-0038-42), CellTrace CFSE Cell Proliferation Kit (catalog C34554), and 6x-His Tag mAb/FITC (catalog MA 1-81891) were purchased from Invitrogen. APC conjugation kit lighting link (catalog ab201817) was purchased from Abcam and was used to label 6B1 according to manufacturer’s instruction. CD antibodies: CD15 (clone H198), CD33(clone P67.6), and CD45RA (clone HI100) were purchased from BioLegend. Mouse mAb to HLA class I (W6/32) was obtained from the MSKCC Monoclonal Antibody Core Facility. Human isotype control hIgG1 antibody was purchased from Bingo Biotech (catalog ET901). CytoTox 96 Nonradioactive cytotoxicity assay kit (catalog G1780) was obtained from Promega. Human IFN-γ was purchased from R&D Systems. Dynabeads human T activator CD3/CD28 was purchased from Thermo Fisher Scientific.

### Peptides

All peptides were purchased and synthesized by Genemed Synthesis Inc. Peptides were > 95% pure (Tables 1 and 2). The peptides were dissolved in dimethyl sulfoxide and diluted in saline at 5 mg/mL and frozen at −80°C.

### Flow cytometry analysis

For cell surface staining, cells were incubated with appropriate mAbs for 30 minutes on ice, washed, and incubated with secondary antibody reagents when necessary.

Live cells were gated based on forward scatter (FSC) and side scatter (SSC), and then unstained cells were used as negative control. All groups had the same gate; each group was overlaid as histograms for comparison. Unstained cells and control mAb staining were shown as controls. Flow cytometry data were collected on a LSR Fortessa (BD Biosciences) and analyzed with FlowJoV10.6.1 software.

### EBV-specific T cell expansion

T cells were enriched from PBMCs by depletion of monocytes by adhesion. Nonadhering cells were stimulated with irradiated autologous EBV-transformed B cells (EBV-BLCLs) generated by transformation with the B95.8 strain of EBV at a 20:1 responder/stimulator (R/S) ratio and cultured in RPMI1640 medium, containing 10% human serum (HS). Beginning on day 7, IL-2 at 20 to 80 units/mL (Collaborative Biomedical Products) and IL-15 at 10 ng/mL (NCI) were added to the T cell cultures every 2–3 days and were restimulated weekly with the same EBV-BLCLs at a 4:1 R/S ratio.

### Validation of the targets

#### T cell response.

Immunogenicity of the pIRS2 peptide was validated using T cell response as surrogate. PBMCs from HLA-A*02:01 healthy donors were obtained by Ficoll density centrifugation. CD14^+^ monocytes were isolated and used for antigen presenting cells (APCs) and for DC generation to stimulate T cells in vitro. CD3^+^ T cells were isolated by negative immunomagnetic cell separation using a pan T cell isolation kit. T cells were stimulated with autologous APCs for 3–4 rounds, and peptide-specific response was determined by IFN-γ ELISpot, as described previously (34, 35).

#### MS.

pIRS2 epitope presented by HLA-A2 molecule was detected by MS as described previously (36) in thyroid cancer cell line TPC-1, breast cancer cell line MDA-MB-231, Burkitt lymphoma cell lines Jeko and NCEB1, and ALL cell line BV173, without specific phosphopeptide enrichment, suggesting a relatively high density of the epitope on the cell surface. Suspension cells were harvested through direct resuspension, and adherent cell lines were harvested after incubating 15 minutes with CellStripper solution (Corning, catalog 25056CI). Harvested cells were pelleted and washed 3 times in ice-cold sterile PBS (Media Preparation Core, MSKCC). In total, 20 million cells were used per experiment. Cells were lysed in 7.5 mL of 1% CHAPS (MilliporeSigma, catalog C3023) dissolved in PBS and supplemented with protease inhibitors (cOmplete, Roche, catalog 11836145001). Cell lysis was performed for 1 hour at 4°C, lysates were spun down for 1 hour with 20,000*g* at 4°C, and supernatant fluids were isolated.

A total of 40 mg of cyanogen bromide-activated Sepharose 4B (MilliporeSigma, catalog C9142) was activated with 1 mM hydrochloric acid (MilliporeSigma, catalog 320331) for 30 minutes.

Subsequently, 0.5 mg of W6/32 antibody (Bio X Cell, catalog BE0079) were coupled to Sepharose in the presence of binding buffer (150 mM sodium chloride, 50 mM sodium bicarbonate [pH 8.3], sodium chloride [MilliporeSigma, catalog S9888], sodium bicarbonate [MilliporeSigma, catalog S6014]) for at least 2 hours at room temperature. Sepharose was blocked for 1 hour with glycine (MilliporeSigma, catalog 410225) and washed 3 times with PBS.

Supernatants of cell lysates were run over the different types of columns through peristaltic pumps (Pharmacia Biotech, Model P-1) with 1 mL/min flow rate overnight in a cold room. Affinity columns were washed with PBS for 30 minutes and in water for 30 minutes; they were then run dry, and HLA complexes were subsequently eluted 5 times with 200 μL 1% TFA (MilliporeSigma, catalog 02031).

For separation of HLA ligands from the HLA complexes, C18 columns (Sep-Pak C18 1 cc Vac Cartridge, 50 mg sorbent per cartridge, 37–55 μm particle size; Waters, catalog WAT054955) were preconditioned with 80% ACN (MilliporeSigma, catalog 34998) in 0.1% TFA and equilibrated with 2 washes of 0.1% TFA. Samples were loaded, washed again with 0.1% TFA, and eluted in 400 μL of 30%, 40%, or 50% ACN in 0.1% TFA. Sample volume was reduced by vacuum centrifugation for MS analysis.

#### Solid-phase extractions.

In-house C18 minicolumns were prepared as follows: for solid-phase extraction of 1 sample, 2 small disks of C18 material (1 mm in diameter) were punched out from CDS Empore C18 disks (Thermo Fisher Scientific, catalog 13-110-018) and transferred to the bottom of a 200 μL Axygen pipette tip (Thermo Fisher Scientific, catalog 12639535). Columns were washed once with 100 μL 80% ACN/0.1% TFA and equilibrated 3 times with 100 μL 1% TFA. All fluids were run through the column by centrifugation (500*g*, 20°C, 10 minutes) in mini tabletop centrifuges, and eluates were collected in Eppendorf tubes. Then, dried samples were resuspended in 100 μL 1% TFA and loaded onto the columns, washed twice with 100 μL 1% TFA, run dry, and eluted with 50 μL 80% ACN/0.1% TFA. Sample volume was reduced by vacuum centrifugation on a GeneVac evaporator at 500*g*, at 33°C for 2 hours.

#### Liquid chromatography–tandem MS analysis of HLA ligands.

Samples were analyzed by high-resolution/high-accuracy liquid chromatography–tandem MS (LC-MS/MS) (Lumos Fusion, Thermo Fisher Scientific). Peptides were separated using direct loading onto a packed-in-emitter C18 column (75 μm ID/12 cm, 3 μm particles, Nikkyo Technos Co. Ltd.). The gradient was delivered at 300 nL/min, increasing linearly from 2% buffer B (0.1% formic acid in 80% ACN)/98% buffer A (0.1% formic acid) to 30% buffer B/70% buffer A, over 70 minutes. MS and MS/MS were operated at resolutions of 60,000 and 30,000, respectively.

Only peptides with charge states 1, 2, and 3 were allowed. The isolation window was chosen as 1.6 thomsons, and collision energy was set at 30%. For MS/MS, maximum injection time was 100 ms with an automatic gain control of 50,000.

#### MS data processing.

MS data were processed using Byonic software (version 2.7.84, Protein Metrics) through a custom-built computer server equipped with 4 Intel Xeon E5-4620 8-core CPUs operating at 2.2 GHz and 512 GB physical memory (Exxact Corporation). Mass accuracy for MS1 was set to 6 ppm and to 20 ppm for MS2. Digestion specificity was defined as unspecific, and only precursors with charges 1, 2, and 3 and up to 2 kDa were allowed. Protein FDR was disabled to allow complete assessment of potential peptide identifications. Oxidization of methionine; phosphorylation of serine, threonine, and tyrosine; and acetylation of N-terminal were set as variable modifications for all samples. Samples were searched against UniProt Human Reviewed database (20,349 entries, http://www.uniprot.org, downloaded June 2017) with common contaminants added. Peptides were selected with a minimal log probability value of 2, indicating *P* values for peptide spectrum matches of less than 0.01 and duplicates removed.

#### Western blot analysis.

Cell lysates were generated using RIPA buffer with protease and phosphatase inhibitor and quantified using the DC protein assay (Bio-Rad). Protein (20–30 μg) was loaded and separated on 4% to 15% gradient SDS/PAGE gels (Bio-Rad). Proteins were transferred to Nitrocellulose membranes (Thermo Fisher Scientific, 88018), which were blocked for 2 hours with 5% milk at room temperature. Immunoblotting was done using pSer1100-IRS2–specific (Thermo Fisher Scientific; PA5-106094) and with anti–IRS-2 (Abcam; EPR904[2]).

Both antibodies were probed at the manufacturer’s recommended dilution overnight at 4°C before using a secondary antibody directly conjugated to HRP for imaging. Blots were reprobed with an anti–GAPDH-HRP direct conjugated antibody (Cell Signaling Technology, 3683) as a loading control.

### Production of pIRS2/HLA-A2 peptide complexes

The method used follows the original protocol established by David Garboczi (37, 38). Briefly, large amounts of soluble MHC class I/peptide complexes were generated by overexpression of HLA-A2 heavy chain (HC) and β2 microglobulin (β2m) as recombinant proteins in *E*. *coli* and subsequent in vitro refolding and assembly in the presence of high concentrations of pIRS2 peptide. Unphosphorylated IRS2 as well as WT1 peptides were used to generate HLA-A2 complexes to be used as counter selection controls. To obtain soluble MHC/peptide complexes, the HC sequence was mutagenized to remove the cytosolic and transmembrane regions. In order to specifically biotinylate refolded, monomeric MHC/peptide complexes, the HC was expressed as a fusion protein containing a specific biotinylation site at the C-terminus (39–41). These short sequences are sufficient for site-specific, enzymatic in vitro biotinylation of a single lysine residue within this sequence using the biotin protein ligase BirA. Size exclusion chromatography (SEC) was used to separate stable complexes from free β2m and biotin in case protein was in vitro biotinylated.

### Screening of phage library and engineering of full-length human IgG1

A proprietary naive, semisynthetic scFv phage display library (42) was screened for human antibodies that bind the pIRS2/HLA-A2 complex by using standard solution phase phage display panning techniques (40, 41). Briefly, the protein complex was incubated with the phage library and captured using streptavidin-coated magnetic beads. Subsequent bead capture, washing, elution, and phage amplification steps were performed for each round of biopanning. Three rounds of panning were completed using amplified pIRS2/HLA-A2 complex binder-enriched phage pools from the previous round of panning as input for subsequent rounds. Each round of panning included a negative selection step against HLA-A2 protein complexed with unphosphorylated IRS2 peptide, the IRS2/HLA-A2 complex.

To identify scFv fragments that showed high specificity for the pIRS2/HLA-A2 complex, single clones from the third round of panning were analyzed for binding to the pIRS2/HLA-A2 protein complex and BSA (as a nonspecific control) by use of ELISA using an anti-M13 phage monoclonal antibody. Monoclonal phage supernatants that showed pIRS2/HLA-A2 complex–specific binding were selected for antibody sequencing and screened for binding to T2 cells pulsed with pIRS2, IRS2, or irrelevant MHC class I phospho-peptides. Variable regions of selected hits were formatted as full-length human IgG1, followed by expression of the antibodies in mammalian cells and purification.

### BLI

The OctetRed system (ForteBio, Pall LLC) was used to determine the binding properties of mAb 6B1. Biotinylated pIRS2/HLA-A2 was captured with streptavidin biosensors, and the binding was monitored in a 2-fold dilution series of 6B1 starting at 30 nM. The experiment was carried out using kinetic buffer (PBS [pH 7.4], 0.01% BSA, 0.002% Tween-20). Response curves were globally fitted to a 1:1 Langmuir binding model to determine values for the association rate constant (*K*-on), dissociation rate constant (*K*-off), and equilibrium dissociation constant (*K_D_*).

#### Characterization of the full-length hIgG1 for the pIRS2A2 complex.

Initially, the specificities of the fully human IgG1mAbs for the pIRS2/A2 complex were determined by staining T2 cells pulsed with or without pIRS2, IRS2, or other HLA-A2 binding irrelevant peptides, followed by secondary goat F(ab′)2 anti-hIgG mAb conjugated to PE or FITC. The fluorescence intensity was measured by flow cytometry. Direct staining was also performed by conjugating the mAbs with fluorophore APC. The same methods were used to determine the binding of the mAb to fresh tumor cells and cell lines.

#### ADCC.

Target cells used for ADCC were T2 cells pulsed with or without pIRS2, IRS2, or irrelevant HLA-A2 binding peptides, and cancer cell lines without peptide pulsing. pIRS2 mAbs or its isotype control human IgG1 at various concentrations was incubated with target cells and fresh PBMCs (as effectors) at different effector/target ratios for 16 hours. The supernatant was harvested, and the cytotoxicity was measured by LDH release assay with CytoTox 96 nonradioreactive kit from Promega following their instructions. Percent cytotoxicity was calculated using following formula: % cytotoxicity = experimental – effector spontaneous – target spontaneous/target maximum – target spontaneous × 100. Cytotoxicity was also measured by standard 5-hour ^51^Cr release assay. ADCC assays were not statistically comparable between experiments because the sources of human cells changed; in individual experiments, means of triplicate results were normalized. In the case of using BisAbs of 6B1, EBV-specific T cells were used as effector cells as described previously (21). For testing cytotoxicity against AML PDXs, the target cells were labeled with CFSE and coincubated with activated T cells (using CD3/CD28 Dynabeads according to manufacturer’s instruction; Thermo Fisher Scientific) in the presence of 6B1 (1+1) mAb or its isotype control (10 μg/mL) at E:T ratio 6:1 overnight. The cells were harvested, washed, and stained with mAbs for CD33 and other AML markers and dead cells and run on flow cytometry. The percentage of reduction of CFSE^+^ cells or CFSE^+^, dead cells over CFSE^+^ cells were determined as percentage of lysis of targets.

### BisAb engineering

All T cell–dependent BisAb designs were based on a human IgG1 scaffold, possessing the L234A/L235A (LALA) mutations to prevent antibody effector function. The L2K single chain variable fragment (scFv) in variable heavy chain–variable light chain (vH-vL) orientation linked via an 18–amino acid linker (GEGTSTGSGGSGGSGGAD) served as the anti-CD3 moiety for T cell recruitment (22). BisAb C2+2 was constructed by fusing L2K scFv sequences to the C-terminus of the HC of a 6B1 IgG1, separated by a 15–amino acid linker (GGGGS×3). BisAb h2+2 was generated by flanking the L2K scFv with 10–amino acid linkers (GGGGS×2) and inserting into the upper hinge region between C220 and D221. For the asymmetric T cell–dependent BisAbs the Fc region was mutated to create a “knob” (T366W) or “hole” (T366S, L368A, and Y407V) for heteromeric assembly of the respective HCs. BisAb 1+1 consists of a 6B1-IgG1 “hole” and a L2K scFv fused to the IgG1 Fc “knob” via a 15–amino acid linker (GGGGS×3) to the upper hinge. Similarly, BisAb h2+1 was constructed by combining a 6B1-IgG1 “hole,” with a 6B1-IgG1 “knob” in which the L2K scFv is inserted into the hinge region via 2 flanking 10–amino acid linkers (GGGGS×2).

The production of all antibodies was carried out at GenScript. Briefly, antibody sequences were generated by gene synthesis and cloned into cytomegalovirus promoter–driven expression vectors. All proteins were expressed by transient cotransfection in HD 293F cells, purified by affinity chromatography, followed by SEC to obtain the desired purity. The purified antibodies were analyzed by SDS-PAGE, Western blot, and HPLC analysis to determine the molecular weight and purity.

### Modeling TCRm binding to phosphopeptide–HLA-A2 complexes

Models of vH and vL fragments of 6B1 were generated from their respective protein sequences by grafting CDRs onto their canonical framework structures using the LYRA webserver (26). The resulting 6B1 model was docked to the previously published crystal structure of the pIRS2/HLA-A2 complex (PDB ID: 3FQX) (29) using the ClusPro 2.0 webserver in antibody mode (43, 44). The top-scoring complexes based on their clustering properties were then manually inspected for models in which CDR loops were in contact with the phosphopeptide-MHC surface. The resulting models were used as templates for initial poses of 6B1 in complex with pIRS2/HLA-A2 and for other phosphopeptides by mutating the bound phosphopeptide to pT4, pKMD, and pRTF in UCSF Chimera using the Dunbrack and SwissSideChain rotamer libraries (45–47). Initial poses of 6B1 in complex with pIRS2/HLA-A2 and other phosphopeptides in complex with HLA-A2 were submitted to the FlexPepDock web server to determine the most likely phosphopeptide conformation for each phosphopeptide in complex with HLA-A2 and 6B1 (27, 48). For each distinct phosphopeptide, the top 10 lowest energy poses, as determined by their Rosetta energy score, were selected among 300 high-resolution models. To compare the energetic favorability of 6B1 binding to each distinct phosphopeptide/HLA-A2 complex, we computed the binding interface energy of the top 10 scoring models for each distinct complex using the InterfaceAnalyzer application (49) implemented in Rosetta 3 (50). All models were visualized in UCSF Chimera with molecular surfaces computed by the MSMS software package (51).

### Statistics

Two-tailed Student’s *t* test was used in all presented statistical analyses.

### Study approval

Written informed consent was received from participants prior to inclusion in the study. After informed consent on MSKCC IRB-approved protocols, PBMCs from HLA-typed healthy donors were obtained by Ficoll density and used fresh

## Author contributions

All authors whose names appear on the submission made substantial contributions to the study for various experiments and method writing. SSM contributed to the acquisition, analysis, and interpretation of data. ZM performed and wrote computational modeling of the mAb recognition of the phosphopeptides. TD and DAS designed the studies and wrote the manuscript. TK contributed tissue culture and ^51^Cr-release assay. MGK contributed mass spectometry. AGK, MAP, TEW, and PJB contributed generation and characterization of phage antibody hits. RJO provided important intellectual content and edited the manuscript. EKN and ICL designed the BisAb formats and ICL edited the manuscript.

## Supplementary Material

Supplemental data

## Figures and Tables

**Table 3:**
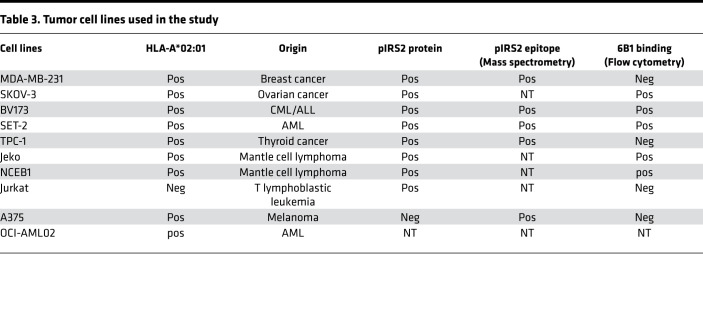
Tumor cell lines used in the study

**Table 2:**
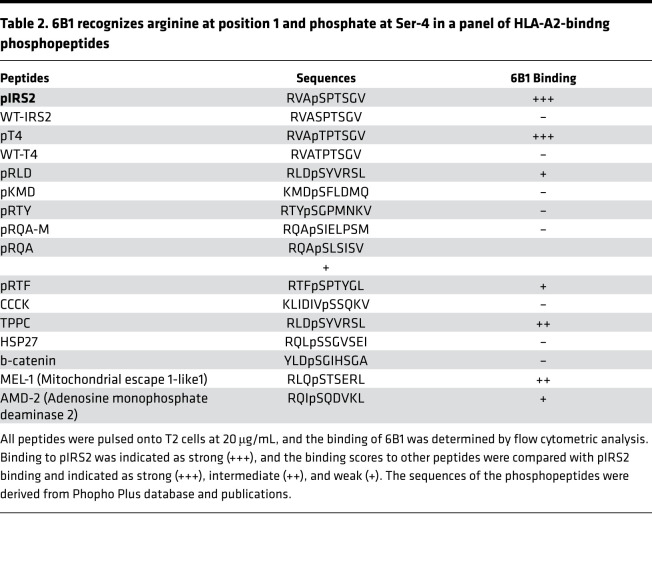
6B1 recognizes arginine at position 1 and phosphate at Ser-4 in a panel of HLA-A2-bindng phosphopeptides

**Table 1:**
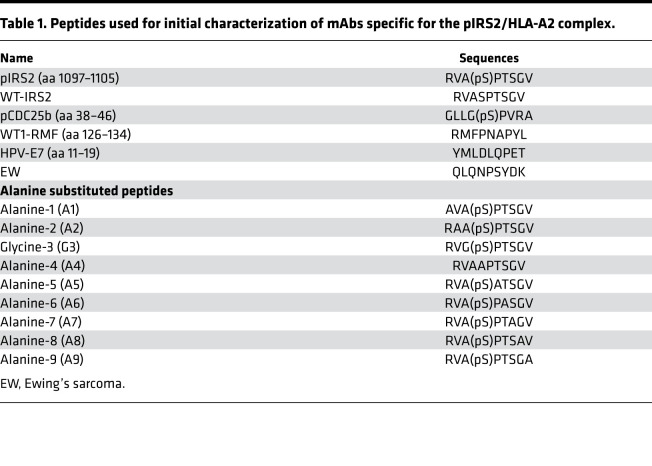
Peptides used for initial characterization of mAbs specific for the pIRS2/HLA-A2 complex.

## Version 1 03/08/2022

Electronic publication

## References

[1] Schumacher T, Schreiber RD (2015). Neoantigens in cancer immunotherapy. Science.

[2] Luksza M (2017). A neoantigens fitness model predicts tumor response to checkpoint blockade immunotherapy. Nature.

[3] McGranahan N (2016). Clonal neoantigens elicit T cell immunoreactivity and sensitivity to immune checkpoint blockade. Science.

[4] Braunlein E, Krackhardt AM (2017). Identification and characterization of neoantigens as well as respective immune responses in cancer patients. Front Immunol.

[5] Tran E (2015). Immunogenicity of somatic mutations in human gastrointestinal cancers. Science.

[6] Carreno BM (2015). Cancer immunotherapy. A dendritic cell vaccine increases the breadth and diversity of melanoma noeantigen-specific T cells. Science.

[7] Finn OJ, Rammensee HG (2018). Is it possible to develop cancer vaccines to neoantigens, what are the major challenges, and how can these be overcome? Nothing new in spite of the name. Cold Spring Harb Perspect Biol.

[8] [No authors listed] (2018). The problem with neoantigen prediction. Nat Biotechnol.

[9] Engelhard VH (2006). Post-translational modifications of naturally processed MHC-binding epitopes. Curr Opin Immunol.

[10] Depontieu FR (2019). Identification of tumor-associated, MHC class II-restricted phosphopeptides as targets for immunotherapy. Proc Natl Acad Sci U S A.

[11] Zarling AL (2006). Identification of class I MHC-associated phosphopeptides as targets for cancer immunotherapy. Proc Natl Acad Sci U S A.

[12] Zarling AL (2000). Phosphorylated peptides are naturally processed and presented by major histocompatibility complex class I molecules in vivo. J Exp Med.

[13] Mohammed F (2008). Phosphorylation-dependent interaction between antigenic peptides and MHC class I: a molecular basis for the presentation of transformed self. Nat Immunol.

[14] Dearth RK (2007). Oncogenic transformation by the signaling adaptor proteins insulin receptor substrate (IRS)-1 and IRS2. Cell Cycle.

[15] Zarling AL (2014). MHC-restricted phosphopeptides from insulin receptor substrate-2 and CDC25b offer broad-based immunotherapeutic agents for cancer. Cancer Res.

[16] Cobbold M (2013). MHC class I-associated phosphopeptides are the targets of memory-like immunity in leukemia. Sci Transl Med.

[17] Engelhard VH (2020). MHC-restricted phosphopeptide antigens: preclinical validation and first-in-juman clinical trial in participants with high-risk melanoma. J Immunother Cancer.

[18] https://investor.agenusbio.com/news-releases/news-release-details/agentus-therapeutics-present-anti-tumor-activities-two.

[19] Dao T (2013). Targeting the intracellular WT1 oncogene product with a therapeutic human antibody. Sci Transl Med.

[20] Chang AY (2017). A therapeutic T cell receptor mimic antibody targets tumor-associated PRAME peptide/HLA-I antigens. J Clin Invest.

[21] Dao T (2015). Therapeutic bispecific T-cell engager antibody targeting the intracellular oncoprotein WT1. Nat Bio Tech.

[22] Brischwein K (2006). MT110: a novel bispecific single chain antibody construct with high efficacy in eradicating established tumor. Mol Immunol.

[23] Ellerman D (2019). Bispecific T-cell engagers: towards understanding variables influencing the in virto potency and tumor selectivity and their modulation to enhance their efficacy and safety. Methods.

[24] Santich BH (2020). Interdomain spacing and spatial configuration drive the potency of IgG-[L]-scFv T cell bispecific antibodies. Sci Transl Med.

[25] Hsiue EHC (2021). Targeting a neoantigen derived from a common *TP53* mutation. Science.

[26] Klausen MS (2015). A webserver for lymphocyte receptor structural modeling. Nucleic Acids Res.

[27] London N (2011). Rosetta FlexPepDock web server--high resolution modeling of peptide-protein interactions. Nucleic Acids Res.

[28] Lazaridis T, Karplus M (1000). Effective energy function for proteins in solution. Proteins.

[29] Petersen J (2009). Phosphorylated self-peptides alter human leukocyte antigen class I-restricted antigen presentation and generate tumor-specific epitopes. Proc Natl Acad Sci U S A.

[30] Mohammed F (2017). The target identity of human class I MHC phosphopeptides is critically dependent upon phosphorylation status. Oncotarget.

[31] Lowe KL (2019). Novel TCR-based biologics: mobilizing T cells to warm “cold” tumors. Cancer Treat Rev.

[32] Sewell AK (2012). Why must T cells be cross-reactive?. Nat Rev Immunol.

[33] Douglass J (2021). Bispecific antibodies targeting mutant *RAS* neoantigens. Sci Immunol.

[34] May R (2007). CD4+ peptide epitopes from WT1 oncoprotein stimulate CD4+ and CD8+ T cells that recognize and kill human malignant mesothelioma tumor cells. Clin Cancer Res.

[35] Pinilla-Ibarz J (2006). Improved human T-cell responses against synthetic HLA-A0201 analog peptides derived from the WT1 oncoprotein. Leukemia.

[36] Klatt MG (2020). Solving an MHC allele-specific bias in the reported immunopeptidome. JCI Insight.

[37] Garboczi DN (1996). Assembly, specific binding, and crystallization of a human TCR-alpha/beta with an antigenic Tax peptide from human T lymphotropic virus type 1 and the class I MHC molecule HLA-A2. J Immunol.

[38] Garboczi DN (1996). Structure of the complex between human T-cell receptor, viral peptide and HLA-A2. Nature.

[39] Altman JD (1996). Phenotypic analysis of antigen specific T lymphocytes. Science.

[40] Busch DH (1998). Coordinate regulation of complex T cell populations responding to bacterial infection. Immunity.

[41] Schatz PJ (1993). Use of peptide libraries to map the substrate specificity of a peptide-modifying enzyme: a 13 residue consensus peptide specifies biotinylation in Escherichia coli. Biotechnology (N Y).

[42] Valadon P (2019). ALTHEA Gold Libraries™: antibody libraries for therapeutic antibody discovery. MAbs.

[43] Desta IT (2020). Performance and its limits in rigid body protein-protein docking. Structure.

[44] Brenke R (2012). Application of asymmetric statistical potentials to antibody–protein docking. Bioinformatics.

[45] Pettersen EF (2014). UCSF Chimera--a visualization system for exploratory research and analysis. J Comput Chem.

[46] Shapovalov MV, Dunbrack RL (2011). A smoothed backbone-dependent rotamer library for proteins derived from adaptive kernel density estimates and regressions. Structure.

[47] Gfeller D (2013). SwissSidechain: a molecular and structural database of non-natural sidechains. Nucleic Acids Res.

[48] Raveh B (2010). Sub-angstrom modeling of complexes between flexible peptides and globular proteins. Proteins.

[49] Lewis SM, Kuhlman B (2011). Anchored design of protein-protein interfaces. PLoS One.

[50] Leaver-Fay A (2011). ROSETTA3: an object-oriented software suite for the simulation and design of macromolecules. Methods Enzymol.

[51] Sanner MF (1996). Reduced surface: an efficient way to compute molecular surfaces. Biopolymers.

