# Supplementary material for: A TCR mimic monoclonal antibody reactive with the “public” phospho-neoantigen pIRS2/HLA-A*02:01 complex
Source: JCI Insight. 2022 Mar 8;7(5):e151624. doi: 10.1172/jci.insight.151624 (PMC8983142; doi:10.1172/jci.insight.151624)
Supplement: Supplemental data [file jciinsight-7-151624-s202.pdf]

Supplementary information for:

A TCR mimic monoclonal antibody reactive with the "public" phospho-neoantigen pIRS2/HLA-A*02:01 complex

[1,5]Tao Dao, [1,5]Sungsoo Mun, [2]Zaki Molvi, [1]Tatyana Korontsvit, [1]Martin G. Klatt, [3]Abdul G. Khan, [3]Elisabeth Nyakatura, [3]Mary Ann Pohl, [3]Thomas White, [3]Paul J. Balderes, [3]Ivo C. Lorenz, [2,4]Richard J. O'Reilly and [1,4] David A. Scheinberg

[1]Molecular Pharmacology Program, Memorial Sloan Kettering Cancer Center, *1275 York Avenue, New York, NY 10065*
[2] Immunology Program, Weill Cornell Medicine, 1300 York Avenue, New York, NY, USA, 10021
[3]Tri-Institutional Therapeutics Discovery Institute, 1300 York Avenue, New York, NY, USA, 10021
[4]Weill Cornell Medicine, 1300 York Avenue, New York, NY, USA, 10021
[5]Equal contribution

Corresponding author:
David A. Scheinberg, MD, PhD
Chair, Molecular Pharmacology Program, SKI;
Memorial Sloan Kettering Cancer Center
1275 York Avenue, New York, NY 10065
Tel. 646-888-2190. scheinbd@mskcc.org

Supplementary figure legends

Figure S1. Target validation. (A) pIRS2 stimulated T cell responses do not show cross-reactivity to the non-phosphorylated peptide (WT-IRS2). Fresh CD3+ T cells from a healthy HLA-A*02:01+ donor were stimulated with pIRS2 peptide 6 times. The peptide-specific response was measured by IFN-gamma ELISPOT assay. pIRS2 is the phosphorylated target peptide and IRS2 is the native cognate peptide. CD14 is normal negative control (autologous CD14+ antigen presenting cells) and EW is an irrelevant HLA-A2-binding peptide. PHA activation with "too numerous to count" spots is used as an internal validation positive control in each experiment. Each point represents average +/- SD from triplicates cultures (*p value* ranges from 0.0015 to 0.00173) and three similar experiments from three different donors. (B). Western blot assays were used to examine expressions of both phosphorylated as well as the native IRS-2 protein in 20-30ug of extracts from indicated cell lines. Blots were derived from replicate samples run on parallel gels. Each blot was probed with either anti-pSer1100-IRS2. (C) MS/MS data from immunoprecipitation with HLA-A, B, C specific antibody in five different cell lines detecting pIRS2 peptides in an A02 and A03 HLA-context.

Figure S2. T cell-mediated cytotoxicity by 6B1 (1+1) against MDA-MB-231, SKOV-3, BV173, Jeko, NCEB1 and SET-2 cell lines was measured by LDH assay, using EBV-T cells at an E:T ratio of 10:1.

Each data point was the average of triplicate cultures plus/minus SD and *P value* ranges are: BV173: 0.013 to 0.025; SKOV3/A2: 0.0019 to 0.02; MDA-MB-231: 0.0017 to 0.0042; Jeko: 0.0061 to 0.04; NCEB1: 0.0075 to 0.03; SET-2: 0.0146 to 0.034. The data are representative of three similar experiments from 3 different donors.

Figure S3. **Interferon-gamma (IFN-γ) enhances the binding of 6B1 mAb to tumor cells.** To test if IFN-γ would enhance HLA-A2 expression and epitope processing, the tumor cell lines MDA-MB-231 (A), SET-2 (B), TPC-1 (C), BV173 (D), and Jurkat (E) were treated with human IFN-γ (100ng/ml) for three days and the binding of the cells to 6B1 mAb and BB7 mAb (anti-HLA-A02) was measured by flow cytometric analysis. IFN-γ treatment significantly enhanced the binding of 6B1 to MDA-MB-231, SET-2, TPC-1 and in a lesser degree to BV173 cells, which was correlated to HLA-A2 upregulation. Jurkat cells are non-HA-A2 and no changes were seen after IFN- **γ** treatment. T2 cells pulsed with pIRS2 peptide (20ug/ml) or irrelevant HLA-A2-binding peptide EW were used as positive and negative controls and experimental groups were indicated in red letters (F). Experimental groups in all other cell lines were indicated in upper and middle right, next to the SET-2 and BV173 cell lines.

Table S1: Inter-residue contact energies between CDRH3 contacts and each phosphopeptide/HLA-A2 complex as determined by Rosetta3 scoring residue contacts of the lowest energy 6B1-phosphopeptide-HLA-A2 models for pIRS2 and pKMD.

Phosphopeptide	CDRH3 Residue	Phosphopeptide residue	Lennard-jones attraction term (kcal/mol)	Lennard-jones repulsion term (kcal/mol)	Lazaridis-Karplus isotropic solvation energy (kcal/mol)
pIRS2	Tyr101	pSer4	-0.149	0.000	0.565
pIRS2	Tyr102	pSer4	-0.006	0.000	0.005
pIRS2	Tyr103	pSer4	-0.049	0.000	0.103
pIRS2	Tyr102	Ser7	-0.006	0.000	0.000
pKMD	Tyr101	pSer4	-0.571	0.000	1.259
pKMD	Tyr101	Phe5	-0.256	0.000	-0.098
pKMD	Tyr102	pSer4	-0.013	0.000	0.059
pKMD	Tyr102	Asp7	-1.054	0.387	1.075

Fig.S1

A

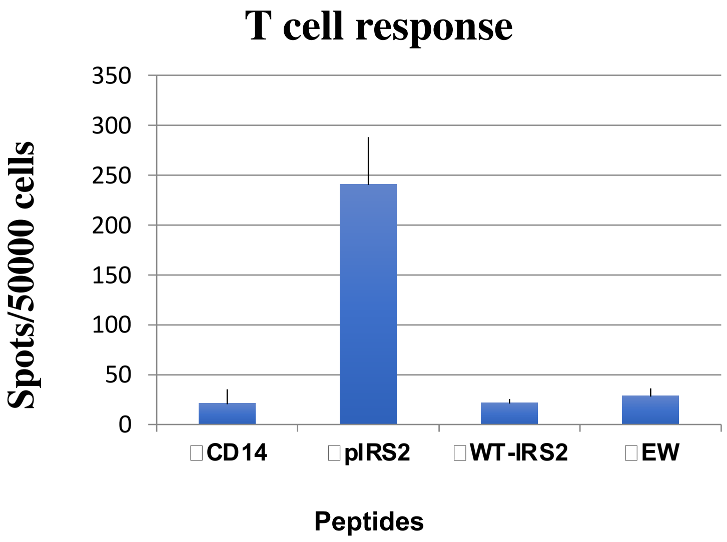

C

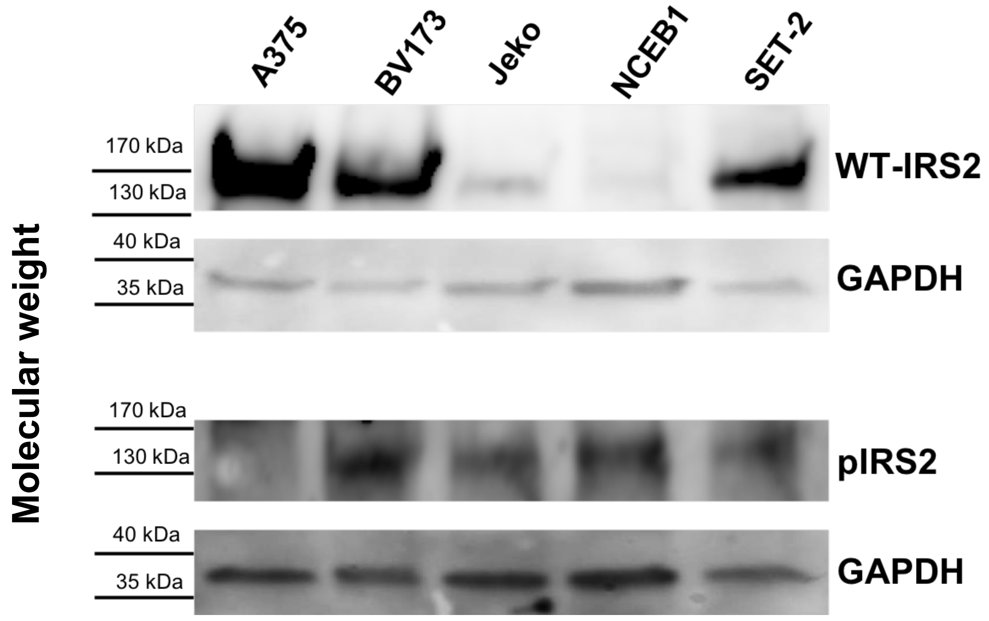

B

sequence with modification	sequence	m/z	charg e	mass error (ppm)	byonic score	log prob	proteins	cell line
A.RVAS[+79.966]PTSGV.K	RVASPTSGV	477.22 6	2	1.3	534.5	4.56	>sp|Q9Y4H2|IRS2_HUMAN Insulin receptor substrate 2 OS=Homo sapiens GN=IRS2 PE=1 SV=2	OCI-AML02
A.RVAS[+79.966]PTSGV.K	RVASPTSGV	477.22 6	2	-0.6	612.7	5.12	>sp|Q9Y4H2|IRS2_HUMAN Insulin receptor substrate 2 OS=Homo sapiens GN=IRS2 PE=1 SV=2	TPC1
A.RVAS[+79.966]PTSGV.K	RVASPTSGV	477.22 6	2	0.3	534.5	4.56	>sp|Q9Y4H2|IRS2_HUMAN Insulin receptor substrate 2 OS=Homo sapiens GN=IRS2 PE=1 SV=2	MDA-MB231
A.RVAS[+79.966]PTSGV.K	RVASPTSGV	477.22 6	2	-0.5	612.7	5.12	>sp|Q9Y4H2|IRS2_HUMAN Insulin receptor substrate 2 OS=Homo sapiens GN=IRS2 PE=1 SV=2	B173
A.RVAS[+79.966]PTSGVK.R	RVASPTSGV K	541.27 5	2	-0.8	487.1	3.76	>sp|Q9Y4H2|IRS2_HUMAN Insulin receptor substrate 2 OS=Homo sapiens GN=IRS2 PE=1 SV=2	U937

Fig. S2

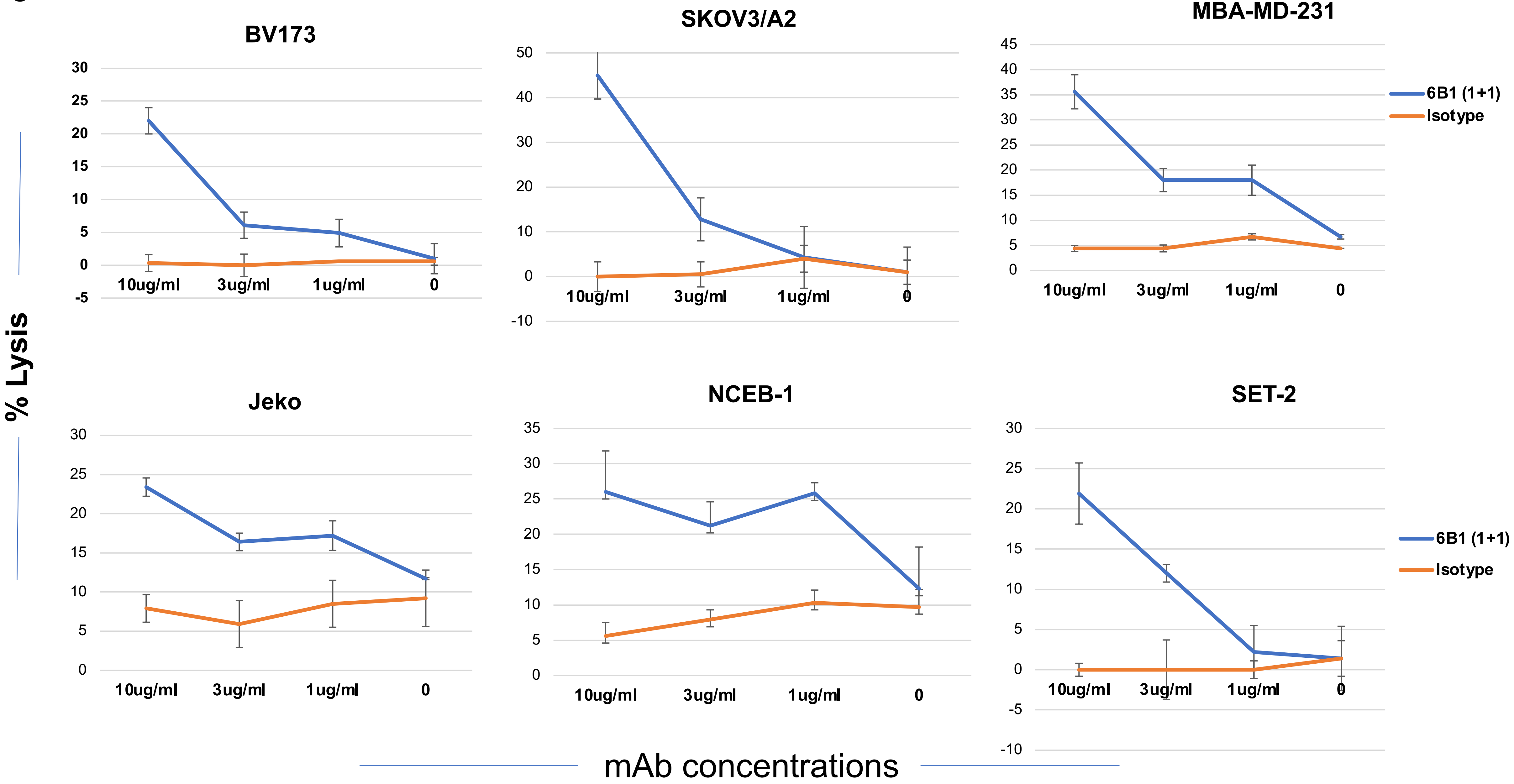
BV173
SKOV3/A2
MBA-MD-231
Jeko
NCEB-1
SET-2
6B1 (1+1)
Isotype
% Lysis
mAb concentrations
10ug/ml
3ug/ml
1ug/ml
0

Fig.S3

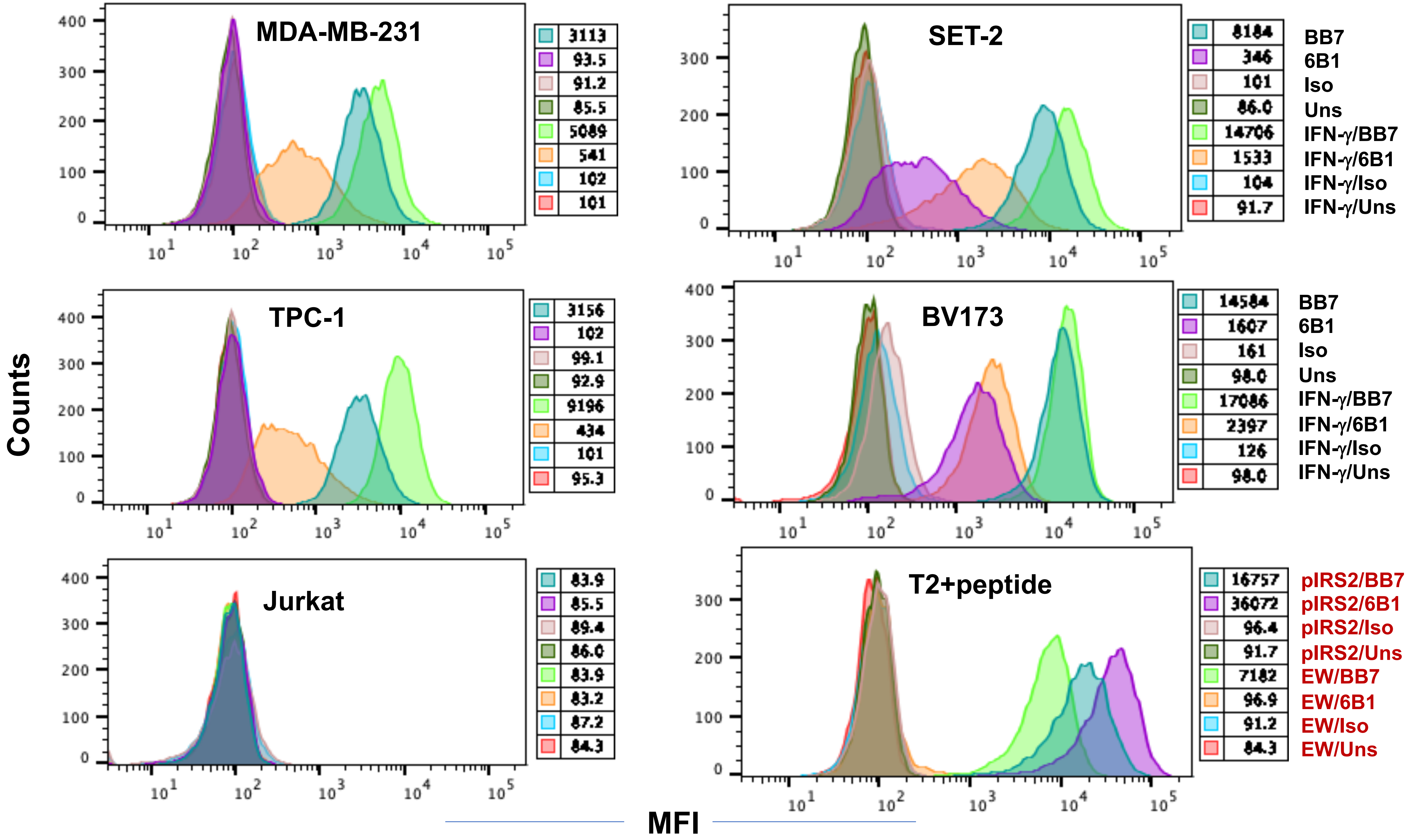
MDA-MB-231
3113
93.5
91.2
85.5
5089
541
102
101
SET-2
8184
346
101
86.0
14706
1533
104
91.7
BB7
6B1
Iso
Uns
IFN-γ/BB7
IFN-γ/6B1
IFN-γ/Iso
IFN-γ/Uns
TPC-1
3156
102
99.1
92.9
9196
434
101
95.3
BV173
14584
1607
161
98.0
17086
2397
126
98.0
BB7
6B1
Iso
Uns
IFN-γ/BB7
IFN-γ/6B1
IFN-γ/Iso
IFN-γ/Uns
Jurkat
83.9
85.5
89.4
86.0
83.9
83.2
87.2
84.3
T2+peptide
16757
36072
96.4
91.7
7182
96.9
91.2
84.3
pIRS2/BB7
pIRS2/6B1
pIRS2/Iso
pIRS2/Uns
EW/BB7
EW/6B1
EW/Iso
EW/Uns
Counts
MFI
